# Replication-Competent Foamy Virus Vaccine Vectors as Novel Epitope Scaffolds for Immunotherapy

**DOI:** 10.1371/journal.pone.0138458

**Published:** 2015-09-23

**Authors:** Janet Lei, Wolfram Osen, Adriane Gardyan, Agnes Hotz-Wagenblatt, Guochao Wei, Lutz Gissmann, Stefan Eichmüller, Martin Löchelt

**Affiliations:** 1 Division of Molecular Diagnostics of Oncogenic Infections, Research Program Infection and Cancer, German Cancer Research Center (DKFZ), Heidelberg, Germany; 2 Division of Translational Immunology, Research Program Tumor Immunology, German Cancer Research Center (DKFZ), Heidelberg, Germany; 3 Bioinformatics Core Facility, German Cancer Research Center (DKFZ), Heidelberg, Germany; Deakin University, AUSTRALIA

## Abstract

The use of whole viruses as antigen scaffolds is a recent development in vaccination that improves immunogenicity without the need for additional adjuvants. Previous studies highlighted the potential of foamy viruses (FVs) in prophylactic vaccination and gene therapy. Replication-competent FVs can trigger immune signaling and integrate into the host genome, resulting in persistent antigen expression and a robust immune response. Here, we explored feline foamy virus (FFV) proteins as scaffolds for therapeutic B and T cell epitope delivery in vitro. Infection- and cancer-related B and T cell epitopes were grafted into FFV Gag, Env, or Bet by residue replacement, either at sites of high local sequence homology between the epitope and the host protein or in regions known to tolerate sequence alterations. Modified proviruses were evaluated *in vitro* for protein steady state levels, particle release, and virus titer in permissive cells. Modification of Gag and Env was mostly detrimental to their function. As anticipated, modification of Bet had no impact on virion release and affected virus titers of only some recombinants. Further evaluation of Bet as an epitope carrier was performed using T cell epitopes from the model antigen chicken ovalbumin (OVA), human tyrosinase-related protein 2 (TRP-2), and oncoprotein E7 of human papillomavirus type 16 (HPV16E7). Transfection of murine cells with constructs encoding Bet-epitope chimeric proteins led to efficient MHC-I-restricted epitope presentation as confirmed by interferon-gamma enzyme-linked immunospot assays using epitope-specific cytotoxic T lymphocyte (CTL) lines. FFV infection-mediated transduction of cells with epitope-carrying Bet also induced T-cell responses, albeit with reduced efficacy, in a process independent from the presence of free peptides. We show that primate FV Bet is also a promising T cell epitope carrier for clinical translation. The data demonstrate the utility of replication-competent and -attenuated FVs as antigen carriers in immunotherapy.

## Introduction

Viral vaccines traditionally consist of attenuated or inactivated viral particles, sub-viral or virus-like particles, or of protein components derived from pathogenic viruses. The purpose of a vaccine is to mount B or T cell memory responses that protect against subsequent pathogen attacks [1]. These responses are often enhanced when antigens are engineered into replication-competent (RC) viral vaccine vectors, either as part of an existing viral protein or as an additional protein. Antigens presented in a highly ordered multimeric array of structural proteins tend to be more immunogenic, as particulate antigens are more likely to be recognized by B cells as foreign [2]. Whole viral particles contain pathogen-associated molecular patterns (PAMPs), such as double-stranded or uncapped RNA, that trigger signaling pathways through toll-like receptors expressed by dendritic cells, thereby facilitating the activation of antigen-specific T cell responses in draining lymph nodes [3]. PAMPs are also strongly expressed during vector replication in infected cells [2]. Cellular damage caused by viruses and RC vectors may also lead to the expression of danger-associated molecular patterns (DAMPs), further activating innate and adaptive immunity [4]. Comprehensive activation of immune signaling pathways by RC vaccine vectors is a prerequisite for the induction of a multifaceted and durable immune response. Depending on the method of application and the site of vector replication, such immune signaling may even lead to immunity in compartments such as the mucosa [5].

Tumor-derived antigens, except those of viral origin, are often poorly immunogenic due to self-tolerance, making the induction of efficient cancer immunity even more challenging. In fact, infiltration of tumors by autologous T cells has recently been correlated with favorable prognosis, suggesting a therapeutic function for tumor reactive T cells in anti-tumor immunity [6]. Within the cellular compartment of the immune system, CD8+ cytotoxic T cells (CTL) are the major effector cells in adaptive anti-tumor immunity and are capable of direct tumor cell killing. CTLs recognize short peptides that are processed from intracellular proteins by sequential proteolytic degradation steps within the cytosol, finally resulting in the presentation of 8-10mer peptides by MHC I molecules on the cell surface [7]. Recognition of such MHC-peptide complexes by activated specific CTLs cells leads to the destruction of the targeted cell.

Traditionally, biological and chemical adjuvants are used to enhance vaccine potency. Of note, current research has highlighted the suitability of RC viruses or attenuated bacteria as adjuvants [8–11]. The mechanism of tumor cell eradication by oncolytic viruses has recently been attributed to the activation of innate immunity and the creation of a hostile, inflammatory environment, rather than to direct virus-mediated tumor cell lysis [12]. Constant antigen expression and PAMP/DAMP exposure promote somatic hypermutation and antibody affinity maturation in B cells [13]. Furthermore, repeated antigen exposure reinforces immunological memory within both the cellular and humoral immune system against subsequent pathogen exposure.

Foamy viruses (FVs) may provide ideal options as RC vectors for therapeutic immunotherapy of persistent infections and cancer. FVs are retroviruses from the *Spumaretrovirinae* subfamily with several unique features in their replication strategy that differentiate them from other retroviruses [14]. FVs are thought to undergo close coevolution with their hosts. Zoonosis has been described among related but not between distantly related species [15]. Laboratory-derived and cloned FV isolates can establish wild-type (wt) infections in their cognate hosts and are slightly attenuated in related host species [16–18]. Despite three decades of work, FV infection has not been conclusively correlated to any disease. Though FVs integrate into host genomes, they have no preference for transcriptionally active regions, unlike their lentiviral and gammaretroviral counterparts [19–21]. Due to their apathogenicity, broad tropism, capacity to reach high titers *in vitro*, and their ability to persistently express heterologous antigens, FVs appear as an attractive source of vaccine scaffolds for safe and effective immunotherapy approaches [14,22–27].

The suitability of FVs as replication-deficient gene therapy vectors for long-term protein expression has been demonstrated in a dog model [28–30]. Several studies have also shown that antibodies were generated against heterologous B cell epitopes displayed by feline foamy virus (FFV) particles or proteins [17,31,32]. Experimental infections of large animals have not led to vector-related pathogenesis, indicating that FVs are suitable for long-term protein, antigen or epitope expression in host cells [17,27]. The use of RC viral vectors in gene therapy raises important safety concerns. RC vectors can be shed by treated individuals or, in the case of retroviral vectors, cause insertional mutagenesis. FV transmission requires close contact of mucosa or bodily fluids, probably limiting e.g. the spread in zoonotically or therapeutically infected humans. Thus, one should consider RC FV vectors for therapeutic vaccination only, if the therapeutic benefit strongly outweighs potential adverse effects, e.g. insertional mutagenesis. The establishment of persistent infections should resolve problems stemming from antiviral immunity. Pre-existing immunity is also negligible, as it can be generally assumed that only a minor fraction of the human population has had the chance to come into contact with primate foamy virus (PFV)-infected simian temple monkeys, bush meat, or laboratory animals [33–38]. Clinical testing of PFV-based vectors is possible in simians, albeit at the expense of ethical concerns and high costs. However, exploratory evaluation of FV-based vaccine vectors requires a suitable non-primate animal model. Since no native rodent FV has been reported, we previously established an FFV infection model in the cat, enabling animal studies [39]. However, ethical concerns, high costs, and the lack of molecular tools for use in cats are still significant limitations. Finally, from the scientific point of view, the genetic stability of recombinant RC vectors and the stability of heterologous gene expression appear decisive for their successful use as viral vaccine carriers. In fact, introduction of transgenes often reduces viral fitness *in vivo*, which may drive transgene loss to preserve genetic economy.

Here, we explored the suitability of FV-based vectors for potential therapeutic use. B and T cell epitopes were selected *in silico* and introduced into FV proteins. To minimize the risk of modifications with detrimental effect on viral replication and to reduce the genetic burden on the host genome, viral protein sequences were replaced by epitopes with high sequence similarity or into protein regions known to tolerate changes while maintaining functional integrity. While replacements in structural proteins were largely detrimental, the accessory protein Bet allowed modifications based on sequence similarity and in regions of low sequence conservation. T cell epitopes derived from the model antigen ovalbumin (OVA), tyrosinase related protein 2 (TRP-2), and the oncoprotein E7 of high-risk human papilloma virus type 16 (HPV16E7) were efficiently processed from recombinant Bet chimera and presented by MHC I molecules on the surface of transfected target cells, resulting in interferon-gamma (IFNγ) release by epitope-specific cytotoxic T cells (CTLs). Efficient processing of a T cell epitope derived from OVA was observed upon FFV infection, however further vector adaptation or purification to achieve efficient infection of murine cells are necessary before functional testing of this novel vaccination platform in mice is feasible.

## Materials and Methods

### Cell culture

HEK293T (American Type Culture Collection, ATCC; CRL-3216), FeFAB [40], CrFK (ATCC, CCL-944), and KE-R (Depository for Cell Lines in Veterinary Medicine, Federal Research Institute for Viral Animal Diseases, Riems, Germany) were propagated under standard conditions in DMEM containing 10% heat-inactivated fetal calf serum plus antibiotics [40]. CrFK-derived FeFAB (FFV-activated β-galactosidase) cells contain the β-galactosidase gene under the control of the FFV internal promoter [40]. All cells were regularly screened for the absence of mycoplasma and other agents and for cell authenticity (Multiplexion, Immenstaad, Germany).

The C57BL/6-derived tumor cell lines EL4 [41] and RMA [42] were cultured in RPMI 1640 supplemented with Glutamax, 10% FCS, and 1% penicillin-streptomycin. The transfectants E.G7 (EL4 cells expressing chicken ovalbumin) [43], 2F11 (RMA cells expressing the HPV16-derived oncoprotein E7) [44] and RMA/TRP-2 (expressing human tyrosinase related protein 2) (kindly provided by A. Paschen), were grown in the same medium containing 0.8 μg/ml G418 (Gibco). The following antigen-specific CTL lines were used: OVA-specific CTL line [45] recognizing the H2K^b^-restricted OVA-derived epitope aa257-264 (SIINFEKL) [46]; E7-specific CTL line [47], reacting against the E7-derived D^b^-restricted epitope RAHYNIVTF (amino acids, aa, 49–57 [48], and a TRP-2-specific CTL line obtained upon TRP-2-specfic DNA immunization of C57BL/6 mice (Osen, unpublished), recognizing the epitope SVYDFFVWL (aa 180–188) [49]. All CTL lines were expanded *in vitro* upon periodical re-stimulation with the transfectant clones expressing the respective target antigen in CTL medium, according to the protocol described in [50].

### Transfection by calcium phosphate

HEK293T cells were transfected in a 6, 10, or 15 cm dish using a modified calcium phosphate method as previously described [40]. FFV titers were determined by a 1:5 serial titration of FFV-containing supernatants on FeFab cells in 96-well microtiter plates [40]. Titrations were performed in triplicate.

### Nucleofection

EL4 and RMA cells were transfected using the Amaxa Cell Line Nucleofector Kit L (Lonza) according to manufacturer’s instructions using a Nucleofector 2B device.

### Molecular cloning of FFV Bet proviral mutants

pCF-7 has been previously described [40]. All PCR products were amplified using Phusion Proofreading Polymerase (New England Biolabs, Frankfurt am Main, Germany).

Epitopes were identified by screening the Immune Epitope database [51,52]. To introduce T cell epitopes derived from melanoma-associated antigen (MAGE) [53,54], TRP-2, OVA, and HPV16E7, and B cell epitopes derived from HA and V5 into *gag* in pCF-7, half of *gag* was amplified by FFV Gag F and either FFV Gag LLK_MLG R, FFV Gag KVLEYVIKV R, FFV Gag Trp2 R, FFV Gag Ova R, FFV Gag HPV16 R, FFV Gag HA R, or FFV Gag V5 R using pCF-7 as template. The other half of *gag* was correspondingly amplified by FFV Gag R and either FFV Gag LLK_MLG F, FFV Gag KVLEYVIKV F, FFV Gag Trp2 F, FFV Gag Ova F, FFV Gag HPV16 F, FFV Gag HA F, or FFV Gag V5 F using pCF-7 as template. The two corresponding fragments were fused by overlap PCR using FFV Gag F and FFV Gag R, digested with XhoI and XmaI and cloned into correspondingly digested pCF-7.

To introduce the HIV B cell epitope 4E10 epitope [55] into *env* in pCF-7, *env* was amplified by FFV Env A F and either FFV Env AB HIV 4E10 N R or FFV Env AB HIV 4E10 D R and FFV Env B R and either FFV Env AB HIV 4E10 N F or FFV Env AB HIV 4E10 D F using pCF-7 as template. The two corresponding fragments were fused by overlap PCR using FFV Env A F and FFV Env B R. The SalI- and BspEI-digested PCR amplicons were cloned into correspondingly digested pCF-7.

To introduce the HIV B cell epitope 2F5 [56], MAGE-derived T cell epitope IMPKAGLLI, and FIV B cell epitope C8 into the C-terminal half of *env*, *env* was amplified by FFV Env B F and either FFV Env B HIV 2F5 R, FFV Env B FIV C8 R, or FFV Env B IMPKAGLLI R and FFV Env B R and either FFV Env B HIV 2F5 F, FFV Env B FIV C8 F, or FFV Env B IMPKAGLLI F using pCF-7 as template. The two fragments were each fused by overlap PCR using FFV Env B F and FFV Env B R, digested with AcvI and BspEI and cloned into correspondingly digested pCF-7.

To introduce MAGE-derived epitopes into homologous regions in *bet*, *bet* was amplified by FFV Bet F and either R2 Bet sim IMP R, R2 Bet sim FLW R, R2 Bet sim MVK R, R2 Bet sim KVA R, R2 Bet sim SLL R, R2 Bet sim RAL R and FFV Bet R and either R2 Bet sim IMP F, R2 Bet sim FLW F, R2 Bet sim MVK F, R2 Bet sim KVA F, R2 Bet sim SLL F, or R2 Bet sim RAL F using pCF-7 as template. To introduce the well-described TRP2, HPV16E7, Ova- or MAGE-derived epitopes into the C-terminus of *bel2*, *bel1* and *bel2* were amplified by FFV Bet F and either pCF7 Bet-Trp2 R, pCF7 Bet-HPV16 R, Ova8 R, Ova12 R, Ova16 R, Ova20 R, Ova20C5 R, Ova20C10 R, Ova20C15 R, Ova20C20 R, R2 Bet end IMP as, R2 Bet end FLW R, R2 Bet end MVK as, R2 Bet end KVA as, R2 Bet end SLL as, or R2 Bet end RAL as and FFV Bet R and either pCF7 Bet-Trp2 F, pCF7 Bet-HPV16 F, Ova8 F, Ova12 F, Ova16 F, Ova20 F, Ova20C5 F, Ova20C10 F, Ova20C15 F, Ova20C20 F, R2 Bet end IMP s, R2 Bet end FLW F, R2 Bet end MVK s, R2 Bet end KVA s, R2 Bet end SLL s, or R2 Bet end RAL s using pCF-7 as template. The corresponding fragments were fused by overlap PCR using FFV Bet F and FFV Bet R, digested with BspEI and SphI, and cloned into correspondingly digested pCF-7.

### Molecular cloning of subgenomic Bet constructs

pmaxGFP was obtained from Lonza. To introduce TRP2, HPV16E7, and OVA-derived epitopes into *bet*, *bet* was amplified from pBC-Bet or pBC-Bet-Ova8, pBC-Bet-Ova12, or pBC-Bet-Ova20 by Bet max F and either Bet Trp2 R, Bet HPV16 E7 R, Ova8-XhoI R, Ova12-XhoI R, Ova16-XhoI R, or Ova20-XhoI R. Digested PCR products were cloned in pmaxGFP using NheI and XhoI.

### Immunoblotting

To study the cellular steady state protein levels and particle release from 293T cells transfected with vector constructs, HEK293T cells were seeded into 10 cm dishes and transfected with 10 μg vector construct or pCF-7. At 2 d post-transfection (p.t.), cells were lysed in 1% SDS containing protease inhibitor. Nucleic acids were digested using benzonase. Lysates were probed by immunoblotting. FFV Bet was detected by a rabbit anti-Bet polyclonal serum (1:2500) [57]. FFV Gag matrix (MA) was detected by a rabbit anti-Gag MA polyclonal serum (1:3000) [58]. The FFV Env transmembrane (TM) domain was detected by a goat anti-Env TM polyclonal serum (1:1000) [59]. Membranes were incubated with horseradish peroxidase (HRP)-conjugated secondary antibodies (rabbit anti-goat IgG-HRP, Sigma Aldrich, 1:5000; goat anti-rabbit IgG-HRP, Sigma Aldrich, 1:5000) and visualized by enhanced chemiluminescence. Blots were probed against β-actin using a mouse anti-β-actin antibody conjugated to HRP (1:5000, Sigma Aldrich).

### IFNγ enzyme-linked immunospot (ELISpot)

Target cells (EL4 or RMA cells) were infected by culturing 1 x 10^6^ cells in a 6-well dish with 4 mL cleared supernatant from transfected HEK293T cells. Infected cells were harvested 4 d post infection. and served as targets in IFNγ ELISpot assays using 5 x 10^4^ target cells/well as described in [50]. Spots were counted using an AID ELISpot reader (Autoimmun Diagnostika, Strassberg, Germany).

### Bioinformatics

A list of peptide epitopes was retrieved from the Immune Epitope Database on January 11, 2012 [51,52]. Searchable lists were generated using a Perl programming script. Peptide lists were compared to the sequence of FFV proteins Gag, Pol, Env, Bel1, and Bet using FASTA. Secondary structures were predicted using 2DSweep. Surface exposure was predicted using Antigenicity. Binding of peptides to MHC-I and –II was predicted by SYFPEITHI [60]. Sequences were viewed, aligned, and annotated using Geneious Pro 5.4.3.

Data are presented as mean values with standard deviations. Statistical significance was determined using an unpaired two-tailed t-test. Graphs and p-values were calculated using GraphPad Prism 5. * represents p<0.05, ** represents p<0.01, *** represents p<0.001.

## Results

### FFV structural proteins are poor scaffolds for heterologous peptide sequences

**Fig 1:**
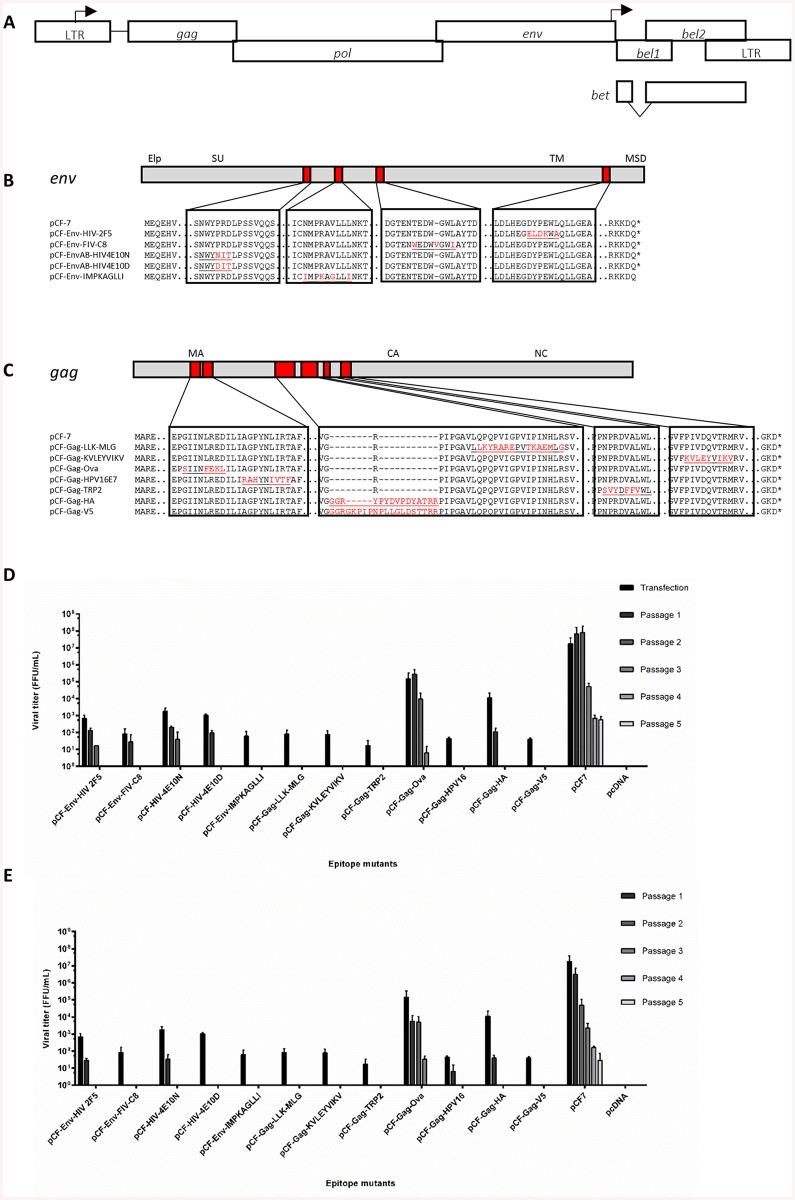
Most modifications in structural proteins are detrimental to viral protein steady state levels and infectivity. (A) Schematic representation of the FFV genome with the long terminal repeats (LTR) and the open reading frames shown as boxes and the promoters in the 5’ LTR and the internal promoter as broken arrows pointing into the direction of transcription. The epitope replacements in Env (B) and Gag (C) are shown below with original protein sequences in black and modifications in red. Epitope sequences are underlined. CrFK (D) and KE-R (E) cells were infected with virus-containing supernatants harvested from 293T cells transfected with wild-type and modified proviruses. Supernatants were serially passaged onto uninfected cells every two days. Viral titers were determined by titration on FeFAB cells. Cells were mock-infected with supernatant from pcDNA-transfected cells as a negative control. Titers are presented as mean values of three independent experiments. Error bars represent standard deviation of mean values.

To evaluate the suitability of RC FFV vectors for the expression of T and B cell epitopes, natural epitopes were compared to the native protein sequence of FFV proteins *in silico*. Replacement of viral sequences by epitopes with similar sequences should minimize disruption of viral functions and preserve viral infectivity. Here, we compared the primary protein sequences of the five known FFV gene products Gag, Pol, Env, Bel1/Tas, and Bet (Fig 1A) to the amino acid sequences of known B and T cell epitopes retrieved from the Immune Epitope Database using FASTA. From the results showing maximal sequence similarity, which was mainly observed for T cell epitopes, we selected epitopes according to their predicted antigenicity using 2DSweep, disease relevance and epitope size. An initial panel of ten T and B cell epitopes as well as the HA and V5 protein tags, were cloned individually into the structural proteins Env and Gag (Fig 1B and 1C) of the FFV-FUV provirus pCF-7.

In Gag, the T cell epitopes LLKYRAREPVTKAEMLG and KVLEYVIKY were grafted by amino acid replacement at the site of highest sequence similarity. T cell epitopes derived from OVA, HPV16E7, and TRP-2 were included to allow further evaluation of CTL activation *in vitro*. The B cell epitope tags HA and V5 were directly inserted into a region of low sequence conservation between different FV Gag proteins. All modifications within Env were performed based on local sequence homology and, in only one case, required the insertion of a single additional amino acid.

Modified proviruses were transfected into HEK293T cells and analyzed after two days for protein expression and particle release. In addition, culture supernatants containing viral particles were serially passaged in permissive CrFK and KE-R cells every two days. Virus titers were determined using the CrFK-derived reporter cell line FeFAB. Since the interval between successive passages is short enough to dilute out even wt FFV (Fig 1D and 1E), this parameter imposed very stringent selection criteria upon the FFV recombinants.

Immunoblotting results demonstrated that recombinants with modifications in Gag showed impaired expression or steady state levels of the 52 kDa Gag precursor protein and its processed 48-kDa form (S1A Fig). Vectors pCF-Gag-HA and -V5 showed slightly larger Gag proteins due to the epitope insertion. Likewise, modifications in Env resulted especially in clones pCF-Env-FIV-C8 and -IMPKAGLII in reduced steady state levels and defective cleavage of the 130 kDa Env precursor into the functional 48 kDa TM protein, and a TM derivative of unknown function [61]. Consequently, FFV particle release of these recombinants was generally severely affected (S1B Fig). Gag-containing FFV particles were only released from chimeric proviruses pCF-Env-HIV-2F5, the highly related vectors pCF-EnvAB-HIV4E10N and -D, pCF-Gag-Ova and, pCF-Gag-HA. Release of Env-only subviral FFV particles [61] was observed for all recombinants except for pCF-Env-FIV-C8 and pCF-Env-IMPKAGLLI, which were both defective in Env processing. Syncytia formation, a hallmark of a productive FFV infection, was absent in all cultures transfected with recombinant provirus, except pCF-Env-HIV-2F5, pCF-Env-HIV-4E10N, pCF-Env-HIV-4E10D, pCF-Gag-Ova and pCF-Gag-HA, correlating with particle release (S1C Fig). Unsurprisingly, FFV titers were drastically reduced in most of the clones (Fig 1D and 1E). Four of the five Env recombinants and two out of seven Gag recombinants could be passaged at least once. Interestingly, pCF-Gag-Ova showed sustained infectivity, albeit at lower level, in both CrFK and KE-R cells, which became evident from the third passage on. Our results show that replacements within the amino acid sequences of the structural proteins Env and Gag are very likely to result in defective or strongly attenuated viruses.

### Bet can host heterologous peptide sequences

Based on the detrimental effects observed with sequence replacements on structural protein function, we tested whether the non-structural Bet protein is similarly sensitive to modifications. Bet is the product of a *bel1-bel2* spliced transcript and counteracts lethal mutagenesis of the viral genome by apolipoprotein B mRNA-editing enzyme 3 (APOBEC3) cytidine deaminases using a pathway distinct from that of lentiviral Vif proteins [58,62–64].

**Fig 2:**
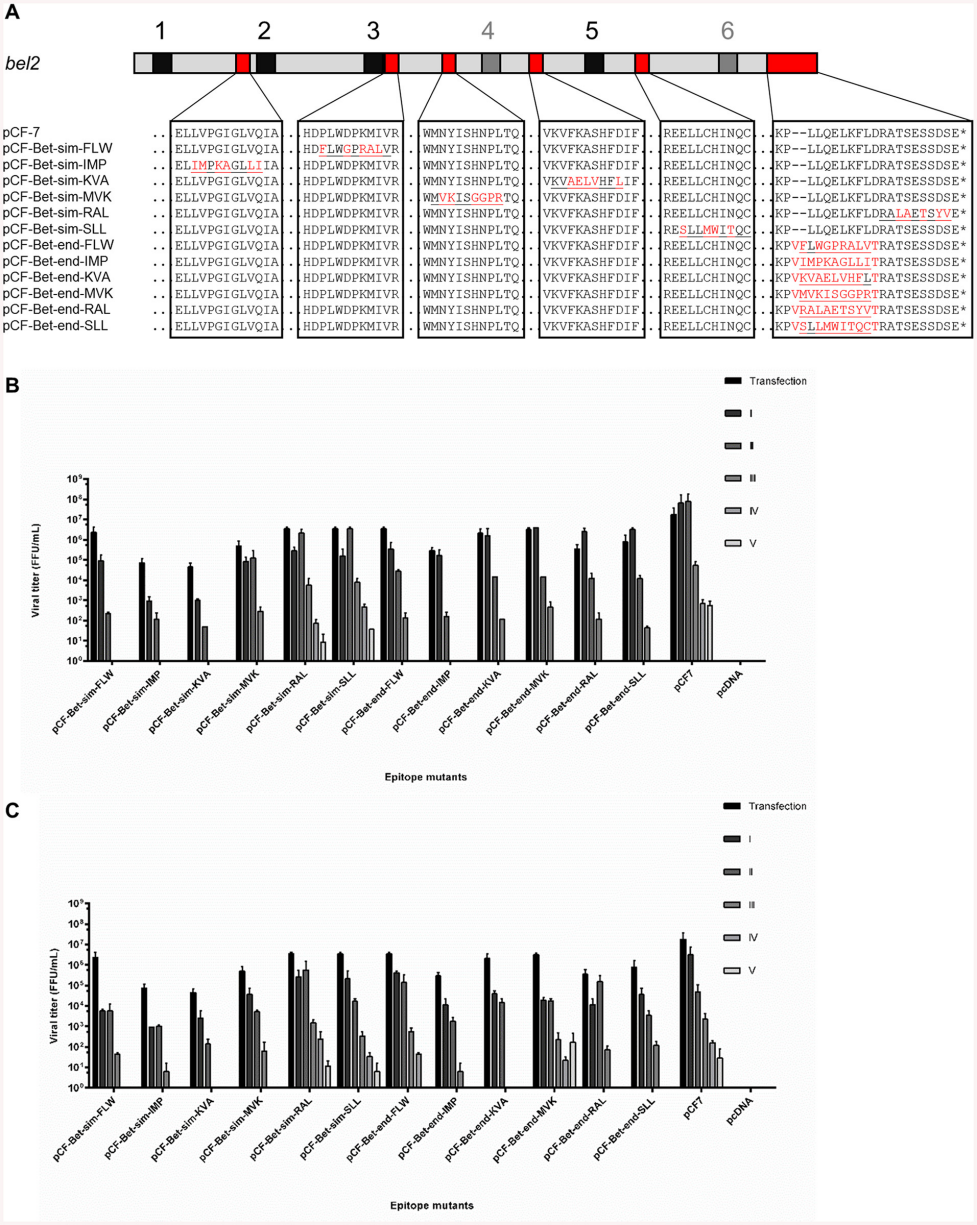
Small modifications in Bet do not affect particle release but may affect FFV titers. A) Schematic representation of epitope replacements in Bet. Conserved motifs are given schematically as black boxes numbered according to [58]. Modifications based on sequence similarity are marked as “sim” and modifications based on position are marked as “end”. Original protein sequences are shown in black; modifications in red. Epitope sequences are underlined. B) CrFK and C) KE-R cells were infected with virus-containing supernatants harvested from 293T cells transfected with modified and wild-type proviruses. Supernatants were passaged onto uninfected cells every two days. Viral titers were determined by titration on FeFAB cells. Cells were mock-infected with supernatant from pcDNA-transfected cells as a negative control. Titers are presented as mean values of three independent experiments. Error bars represent standard deviation of mean values.

We evaluated the effectiveness of the protein sequence similarity-based approach and the approach based on previously identified non-conserved regions that may tolerate sequence changes in hosting different epitopes. A panel of six 9-mer T cell epitopes derived from the MAGE family was selected *in silico* [51,52,65]. Each epitope was inserted into the site of highest sequence homology to FFV Bet to minimize sequence invasiveness (similarity-based recombinants). In addition, each epitope was grafted into the C-terminus of Bet, which has been shown to be amenable to modifications by heterologous sequences (position-based mutants) [58] (Fig 2A). None of the insertions affected the previously identified conserved motifs in Bet [58] (Fig 2A, black boxes 1–6)

Using these two strategies, recombinant proviruses were generated and analyzed for basic viral functionality. The modified vaccine vectors, pCF-7 and empty vector pcDNA were transiently transfected into HEK293T cells using calcium phosphate. Cells were observed 2 d p.t. for syncytia formation. All proviruses induced syncytia formation in transfected 293T cells at levels similar to the wt provirus pCF-7 (S2A Fig).

Immunoblotting analyses of lysates from transfected cells showed that Gag and Env steady state levels and protein processing were mostly not affected by the sequence modifications in Bet (S2A Fig). Moreover, levels of recombinant particle release and syncytia formation were comparable to the wt (S2B and S2C Fig). Serial passaging of mutant virus in feline APOBEC3-expressing CrFK and KE-R cells showed that similarity-based mutations were slightly less detrimental to FFV replication competence than position-based mutations when considering both, virus titers and numbers of serial passages before loss of virus (Fig 2B and 2C). The data clearly indicate that the non-structural protein Bet is a much more promising epitope scaffold than Gag and Env. While similarity-based modifications of Bet allow better retention of viral function, position-based approaches are much more attractive for general, sequence-independent epitope incorporation strategies.

### Small peptides derived from the model antigen ovalbumin can be hosted at the C-terminus of Bet

**Fig 3:**
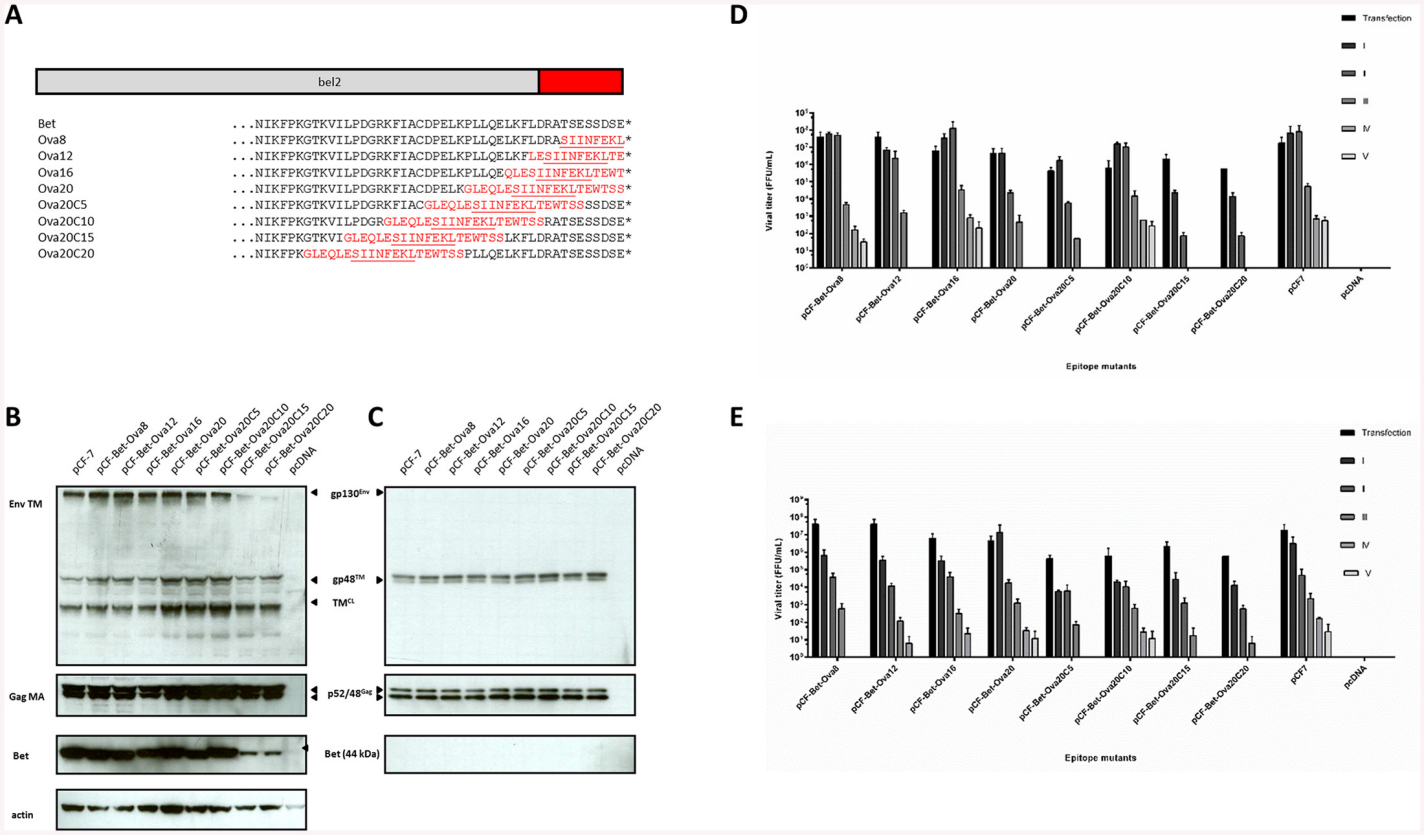
Modifications of FFV *bel2* with OVA do not significantly influence viral protein levels or infectivity. A) Schematic representation of epitope replacements in Bet. Original protein sequences are shown in black; modifications in red. Epitope sequences are underlined. B) Cell lysates and C) enriched culture supernatants of 293T cells transfected with modified and wild-type proviruses were analyzed by immunoblotting. Cells and supernatants were harvested 2 d post-transfection and probed using polyclonal sera against the Gag matrix and Env transmembrane domains. Detection of Env and Gag in the particulate fraction represents specifically released virus particles. The Gag precursor (p52), cleaved mature Gag (p48), Env precursor (gp130Env), mature TM (gp48TM) and a cell lysate-associated transmembrane isoform (TM^CL^) are indicated by arrows. Proper protein loading of cell lysates was determined by probing for β-actin. D) CrFK and E) KE-R cells were infected with virus-containing supernatants harvested from 293T cells transfected with modified and wild-type proviruses. Supernatants were passaged in uninfected cells every two days. Viral titers were determined by titration on FeFAB cells. Cells were mock-infected with supernatant from pcDNA-transfected cells as a negative control. Titers are presented as mean values of three independent experiments. Error bars represent standard deviation of mean values.

To determine whether CTL epitopes grafted into the C-terminus of Bet are capable of being processed and presented to epitope-specific CD8^+^ T cells, we used the H2K^b^-restricted CTL epitope SIINFEKL, derived from the well-characterized model antigen chicken ovalbumin (OVA) [46]. The FFV provirus pCF-7 was modified by replacement of C-terminal amino acids of Bet by different OVA-derived sequences. MHC-I-restricted epitope presentation depends not only on delivery of the antigen into the cell, but also on efficient processing of cellular and viral proteins by the proteasome. Therefore, we constructed mutants with flanking sequences of various lengths derived from the native primary structure of the OVA protein encompassing the SIINFEKL epitope (Fig 3A). HEK293T cells were transfected with FFV Bet-Ova vectors to determine protein expression, particle release, and infectivity. Viruses containing modifications close to the C-terminus showed comparable levels of Gag and Env expression, processing, and particle release compared to wt pCF-7 (Fig 3B and 3C). However, Bet chimera Ova20C15 and Ova20C20, in which the 20-mer epitope extends 35 and 40 amino acids into the N terminus of Bet, respectively, showed decreased steady state Bet protein levels. Serial passaging in CrFK and KE-R cells revealed few or no differences for most of the chimera compared to the wt (Fig 3D and 3E). However, titers of Ova20C15 and Ova20C20 were negatively impacted. Since substantial Bet expression is required for efficient APOBEC3 inactivation [58], decreased Bet steady levels of these FFV-Ova chimera, as observed in Fig 3B, likely caused attenuated replication in APOBEC3-positive CrFK and KE-R cell lines. Since both epitope insertions strongly affect conserved motif 6 in FV Bet (Fig 2A) [58], this part of Bet may be required for protein stability or function. In summary, the data show that only proviruses with C-terminal replacements in Bet retain full replication competence.

### FFV Bet can host other therapeutic T cell epitopes

While OVA represents a widely used model antigen, we also examined proteins of clinical relevance. We tested whether T cell epitopes derived from the melanoma-associated antigen TRP-2 and the high-risk HPV16 oncoprotein E7, respectively, could be functionally integrated at the C-terminus of Bet. We also investigated whether the epitopes were efficiently processed and presented to specific T cells. TRP-2 is a tautomerase involved in the melanin biosynthesis pathway and harbors the H2-K^b^-restricted CTL epitope TRP-2_180–188_ (SVYDFFVWL) [49]. HPV16 is a high-risk mucosal HPV type associated with the development of cervical cancer [66]. The E7 oncoprotein binds to the retinoblastoma protein pRb and, in tandem with the second HPV oncoprotein, E6, promotes cell cycle progression in infected cells [67–69]. Priming of the adaptive immune system in C57BL/6 mice with the H2-D^b^-restricted, E7-specific CTL epitope E7_49-57_ (RAHYNIVTF) protected mice against outgrowth of HPV16-transformed tumor cells [48].

**Fig 4:**
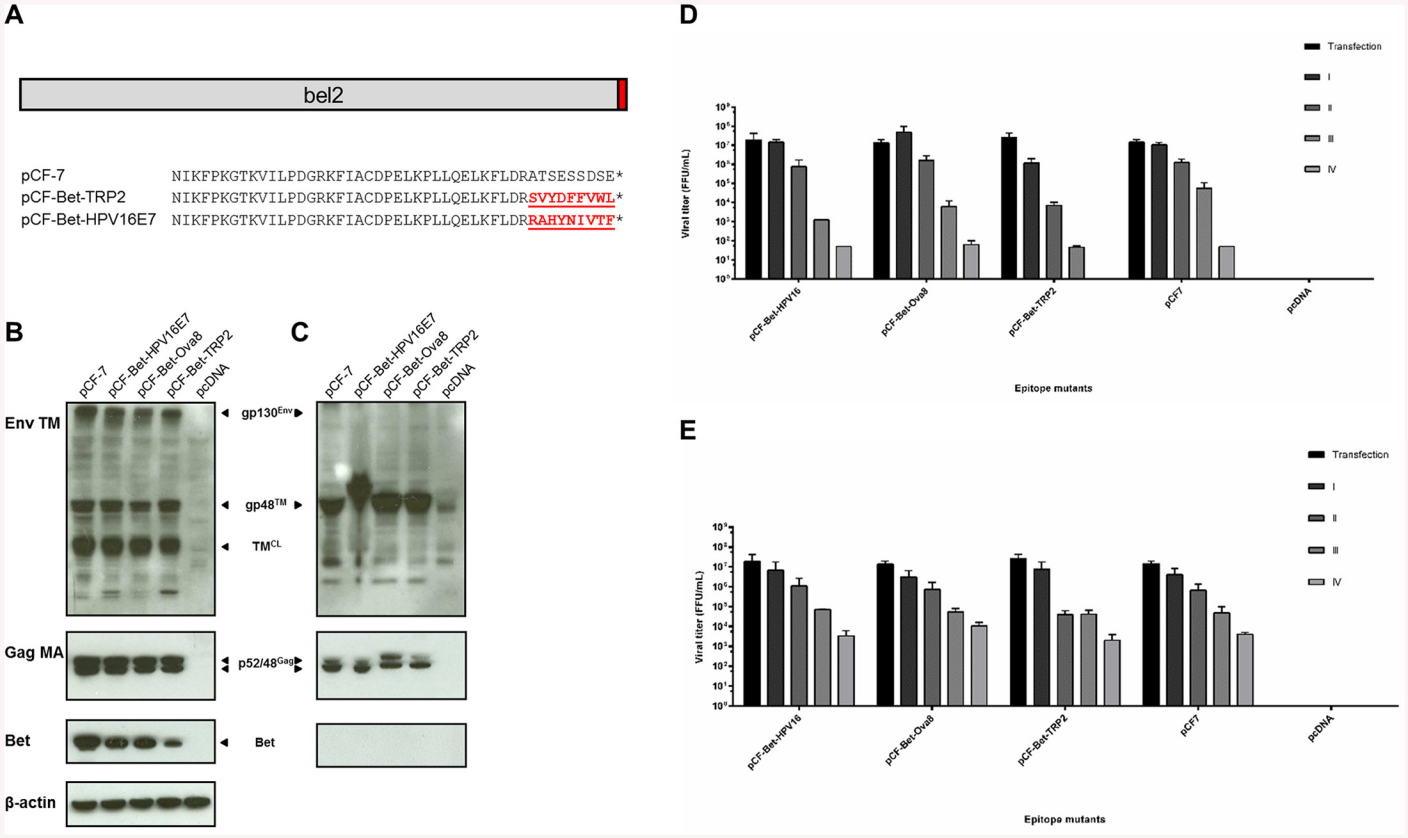
Modification of FFV *bel2* by TRP2 and HPV16E7 epitopes does not significantly affect viral functions. A) Two different 9-mers derived from TRP-2 and HPV16E7 were cloned into the C-terminus of Bet. Original protein sequences are shown in black and modifications are shown in red. Epitope sequences are underlined. B) Cell lysates and C) enriched culture supernatants of 293T cells transfected with pCF-7, pCF-Bet-Ova8, pCF-Bet-HPV16E7, pCF-Bet-TRP2 and pcDNA were analyzed by immunoblotting 2 d post-transfection and probed using polyclonal sera against the Gag matrix and Env transmembrane domains. Detection of Env and Gag in the particulate fraction represents specifically released virus particles. The Gag precursor (p52), cleaved mature (MA) Gag (p48), Env precursor (gp130Env), mature TM (gp48TM) and a cell lysate-associated transmembrane isoform (TM^CL^) are indicated by arrows. Proper protein loading of cell lysates was determined by probing for β-actin. Feline APOBEC3 expressing D) CrFK and E) KE-R cells were infected with virus-containing supernatants harvested from 293T cells transfected with modified and wild-type proviruses. Supernatants were passaged in uninfected cells every two days. Viral titers were determined by titration on FeFAB cells. Cells were mock-infected with supernatant from pcDNA-transfected cells as a negative control. Titers are presented as mean values of three independent experiments. Error bars represent standard deviation of mean values.

The T cell epitopes SVYDFFVWL and RAHYNIVTF were grafted into the C-terminus of Bet in the infectious FFV proviral genome (Fig 4A). pCF-7, pCF-Bet-TRP2, or pCF-Bet-HPV16E7 were transfected into HEK293T and assayed as described above. Immunoblotting showed that Env and Gag steady state levels in pCF-Bet-TRP2- or pCF-Bet-HPV16E7-transfected cell lysates were comparable to the wt (Fig 4B). Particle release from transfected cells was also at levels comparable to the wt pCF-7 (Fig 4C). Serial passaging of infectious viral particles showed that the HPV16 E7 vector was comparable to the wt over several passages in both cell types. In contrast, the TRP-2 vector reached wt titers in KE-R cells but showed reduced infectivity in CrFK cells, possibly due to reduced steady state levels of the chimeric Bet protein (Fig 4B, 4D and 4E).

### The OVA-specific, H2-K^b^-restricted T cell epitope SIINFEKL is presented by murine target cells expressing chimeric Bet-epitope fusion proteins

The results presented above show that Bet is a suitable scaffold for the incorporation of different T cell epitopes. Grafting of T cell epitopes into Bet selected from our initial *in silico* screen and because of the availability of required reagents was shown to have little consequence on viral titers. Though PFV and FFV replication has been shown to be restricted in murine cells [70], we nevertheless chose murine target cells and murine CTL lines to test CTL reactivity *in vitro* and promising results could be further examined by validation of PFV-based therapeutic vectors in a primate model. Thus, sub-genomic Bet-Ova constructs were initially employed to analyze the expression, processing, and MHC I-restricted presentation of Bet-engrafted T cell epitopes. The use of Bet expression constructs eliminates the need for FFV-susceptible cells and FFV gene expression in murine target cells.

Recognition of a T cell epitope by CTLs is the end result of intracellular proteolytic cleavage and transport processes, culminating in binding of 8mer to 10mer peptides to MHC I molecules [71]. Using a SIINFEKL-specific CTL line established from splenocytes of immunized C57BL/6 mice, we tested whether the SIINFEKL sequence cloned into the C-terminus of Bet, flanked by sequences of various lengths derived from epitope encompassing regions within the native OVA sequence, would be properly processed and presented by transfected cells. The cDNA sequence of Bet or of epitope-carrying Bet derivatives was cloned into the expression vector pmaxGFP and tested for expression in 293T cells showing proper expression of the chimeric Bet proteins (data not shown). Murine EL4 or RMA cells were transfected with the plasmids by nucleofection and used as target cells in IFNγ ELISpot assays together with the OVA-specific CTL line.

**Fig 5:**
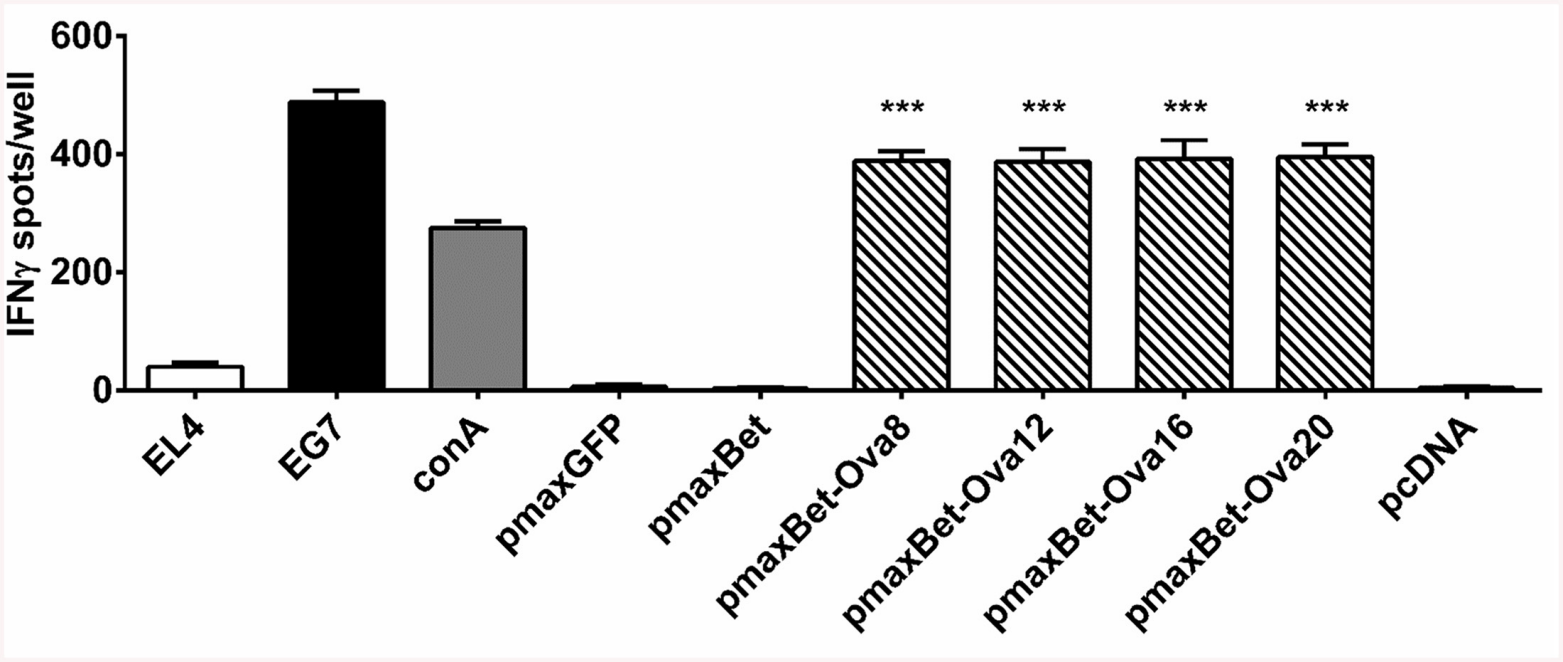
Proper processing and presentation of the OVA-specific H2-K^b^-restricted CTL epitope SIINFEKL from the C-terminus of Bet. EL4 cells nucleofected with different pmaxBet-Ova epitope expression constructs present the H2-K^b^-restricted CTL epitope SIINFEKL on the cell surface, stimulating IFNγ release by SIINFEKL-specific CTLs. Untransfected EL4 cells or EL4 cells transfected with, pmaxGFP, pmaxBet, and pcDNA served as negative controls. The OVA expressing EL4-derived transfectant clone EG7 as well as addition of concanavilin A were used as positive controls. Values represent a single experiment performed in triplicate. *** represents a p-value of less than 0.001 when compared to pmaxBet.

Transfection of EL4 cells with pmaxBet-Ova8, containing the minimal 8-mer epitope, resulted in target cell lines stimulating IFNγ release by the OVA-specific T cell line to similar extend as induced by the OVA-expressing control cell line EG7 or the addition of 10 μg/ml concanavalin A. pmaxBet, lacking the SIINFEKL epitope or pmaxGFP did not induce detectable IFNγ release (Fig 5). These results prove efficient processing and presentation of the CTL epitope from the C-terminus of Bet. The flanking regions introduced into pmaxBet-Ova12, pmaxBet-Ova16, and pmaxBet-Ova20 did not appear to influence the level of IFNγ secretion, showing that their impact on efficient processing of the SIINFEKL epitope from the C-terminus was negligible.

### MHC I-restricted cancer-associated T cell epitopes are presented by murine target cells expressing chimeric Bet-epitope fusion proteins

**Fig 6:**
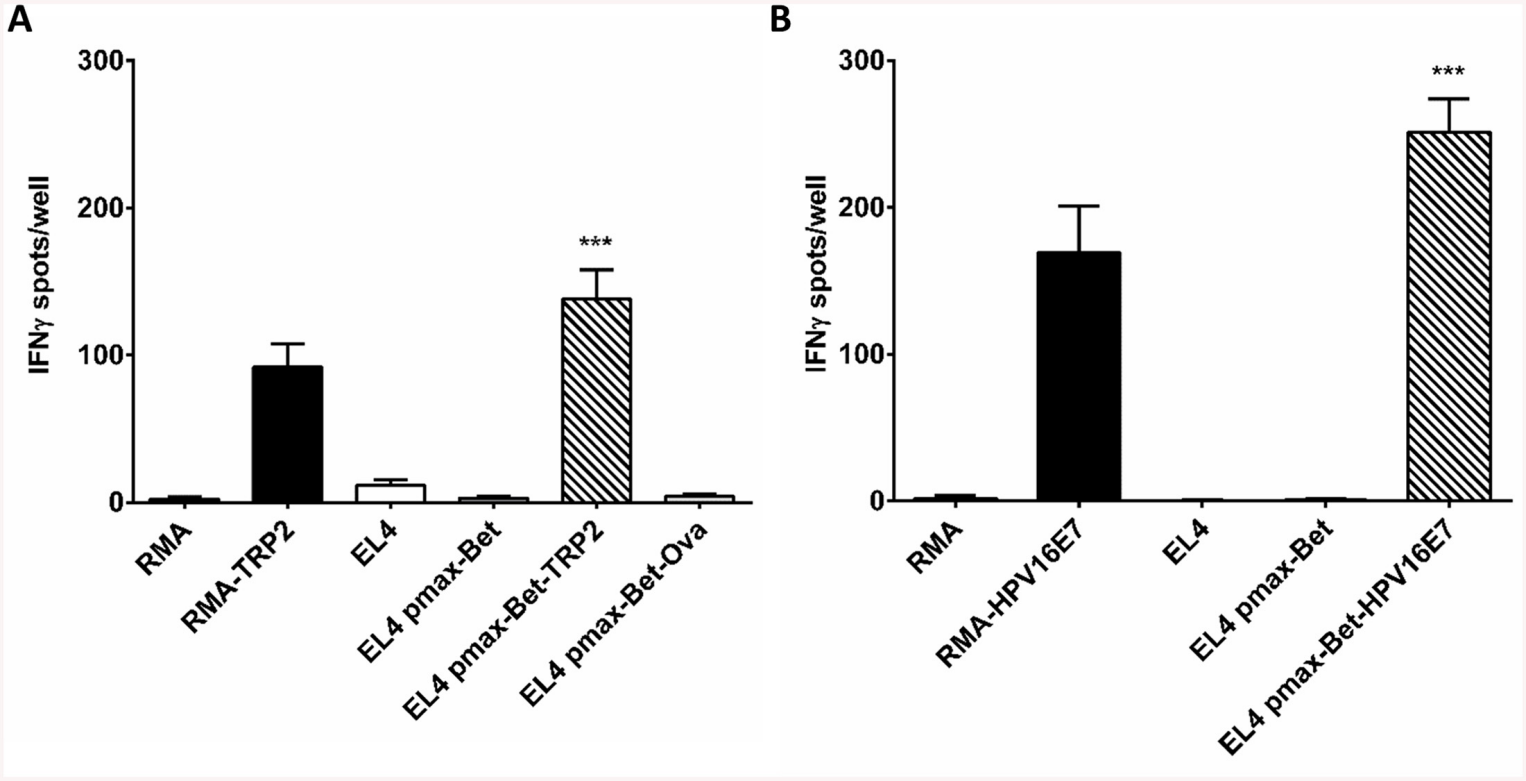
Different tumor antigens are recognized by specific CTLs when placed at the C-terminus of Bet. A) EL4 cells were transfected with pmaxBet or pmaxBet-TRP2 and tested after 2 d with an established TRP-2-specific CTL line in IFNγ ELISpot assays. The stably TRP2-expressing cell line RMA-TRP2 was used as positive control. EL4 and RMA cells either untransfected, or transfected with pmaxBet or pmaxBet-Ova8 were used as negative controls. Results are shown as number of IFNγ spots per 12500 CTLs/well. B) EL4 cells were transfected with pmaxBet or pmaxBet-HPV16E7 and tested after 2 d with E7-specific CTLs in an IFNγ ELISpot assay. The stable HPV16E7-expressing cell line RMA-HPV16E7 was used as positive control. Untransfected EL4 and RMA cells and pmax-Bet-transfected EL4 cells were used as negative controls. Results are shown as number of IFNγ spots per 625 CTLs/well. Values represent a single experiment performed in triplicate. ** represents a p-value of less than 0.01 when compared to pmaxBet; ***, p-value < 0.001.

The DNA sequences encoding the T cell epitopes TRP-2_180–188_ and E7_49-57_ were cloned into the C-terminus of Bet to obtain the expression vectors pmaxBet-TRP2 and pmaxBet-HPV16E7, respectively. EL4 cells were transfected by nucleofection with pmaxBet-TRP2, pmaxBet-HPV16E7, or pmaxBet. Cells were harvested two days later and co-cultured with TRP-2-specific or with E7-specific CTL lines, respectively, in IFNγ ELISpot assays. EL4 cells transfected with pmaxBet-TRP2 induced maximal IFNγ secretion among TRP-2-specific CTLs, even exceeding the magnitude of IFNγ response obtained with RMA/TRP-2 transfectants used as positive control target cell line. Cells transfected with the parental vector pmaxBet or Bet-Ova8 induced only marginal IFNγ secretion (Fig 6A). Likewise, EL4 cells transfected with pmaxBet-HPV16E7 strongly activated of RAHYNIVTF-specific CTLs (Fig 6B), again to greater extent than the control cell line expressing the target antigen endogenously, i.e RMA-HPV16E7.

### PFV Bet can act as an alternative epitope carrier

**Fig 7:**
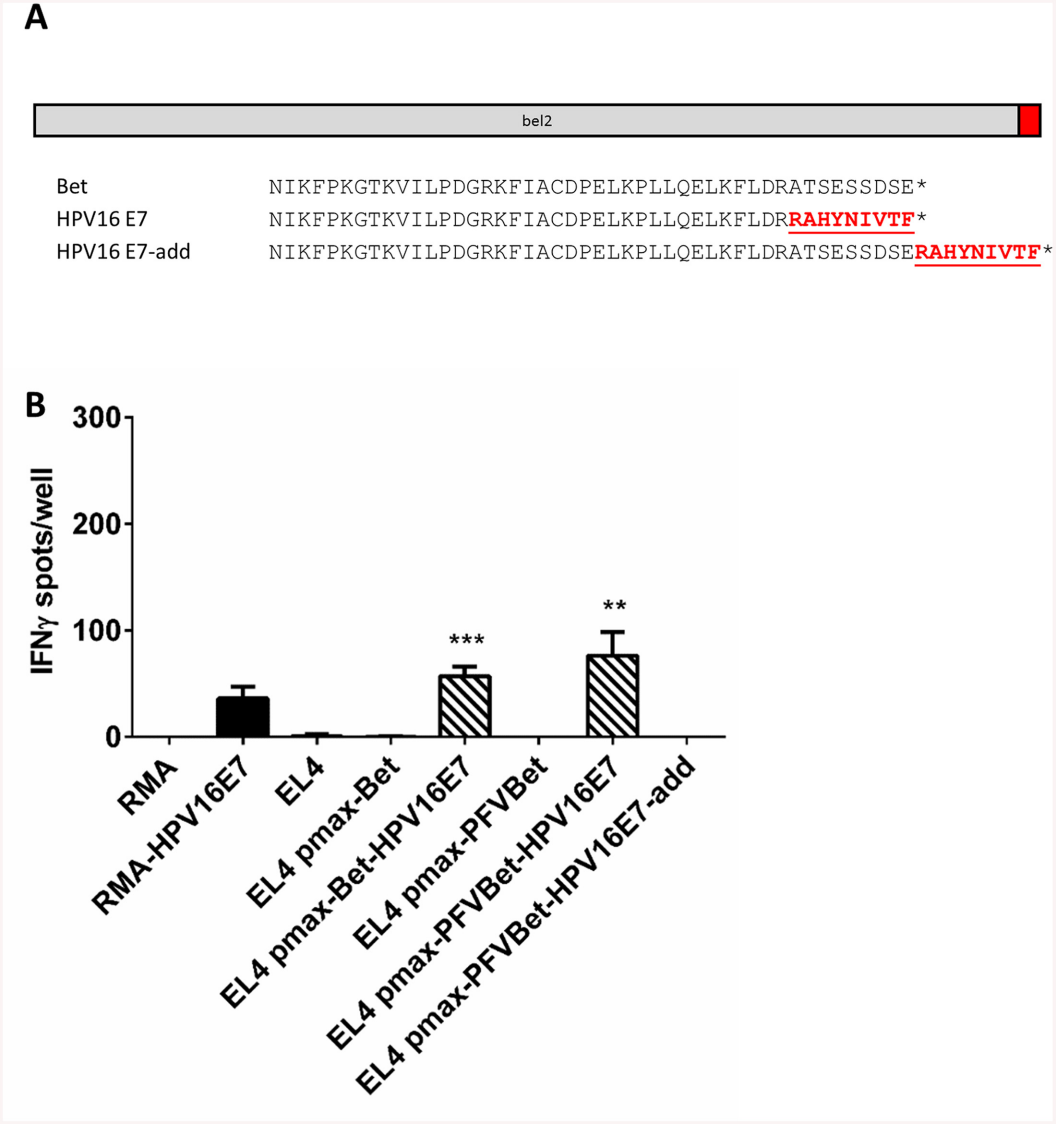
Recognition of the HPV16 E7-derived H2-D^b^-restricted epitope RAHYNIVTF incorporated into the PFV Bet C-terminus by E7-specific CTLs. A) The coding sequence for PFV Bet was cloned into the expression vector pmaxGFP to yield pmaxPFVBet. The HPV16E7 T cell epitope RAHYNIVTF was cloned into the C-terminus of PFV Bet. A replacement of the last nine amino acids of PFV Bet resulted in pmaxPFVBet-HPV16E7. Upon addition of the nine amino acids to the C-terminus of PFV Bet pmaxPFVBet-HPV16E7-add was generated. Original protein sequences are shown in black and modifications are depicted in red. B) EL4 cells were nucleofected with pmaxPFVBet, pmaxPFVBet-HPV16E7, or pmaxPFVBet-HPV16E7-add. After 2 d, cells were harvested and analyzed in an IFNγ ELISpot assay using HPV16E7-specific CTLs. The HPV16 E7-expressing transfectant clone RMA-E7 and RMA cells transfected with the FFV Bet-encoding plasmid pmax-Bet-HPV16E7 were used as positive control. Untransfected EL4 cells and EL4 transfected with FFV and PFV pmaxBet vectors were used as negative controls. Results are shown as number of IFNγ spots per 1250 CTLs/well. Values represent a single experiment performed in triplicate. ** represents a p-value of less than 0.01 when compared to pmaxPFVBet; ***, p-value < 0.001.

Since PFV is a primate virus recovered from a zoonotically infected person and is RC in diverse human cells [37], PFV Bet was also evaluated as T cell epitope carrier. PFV Bet, which has local, limited homology to other non-primate FV Bet proteins including FFV [58], was cloned into the pmax vector by amplification from another cDNA-based expression vector [58] to yield pmaxPFVBet. The nine C-terminal amino acids of PFV Bet were replaced by the HPV16E7-specific T cell epitope RAHYNIVTF to yield pmaxPFVBet-HPV16E7 (Fig 7A). In addition, these nine amino acids were also directly added to the C-terminus of Bet to obtain pmaxPFVBet-HPV16E7-add.

To test whether RAHYNIVTF could be expressed, processed, and presented to H2-D^b^-restricted, E7-specific CTLs, EL4 cells transfected with pmaxPFVBet, pmaxPFVBet-HPV16E7, pmaxPFVBet-HPV16E7-add, and the FFV Bet-based pmax-Bet-HPV16E7 by nucleofection were used as targets in IFNγ ELISpot assays together with the E7-specific CTL line. EL4 cells transfected with pmaxPFVBet-HPV16E7 were able to stimulate IFNγ secretion by E7-specific CTLs more effectively than its FFV Bet-based counterpart (Fig 7B). Cells transfected with pmaxPFVBet-HPV16E7-add or the parental construct pmaxPFVBet did not exhibit any significant CTL activation. These results indicate that the HPV16E7 epitope RAHYNIVTF is capable of inducing IFNγ responses in epitope-specific CTLs when cloned into, but not when added onto the C-terminus of PFV Bet.

### SIINFEKL-encoding FFV infects murine target cells and triggers low levels of IFNγ responses among specific CTLs independent of released SIINFEKL peptides

Although EL4 and RMA cells are not fully susceptible to FFV infection (W. Liu and M. Löchelt, unpublished data) effective T cell epitope presentation may not require a full infection cycle. Transfer of the FV genome and cellular expression of trace amounts of the chimeric Bet-epitope protein could be already sufficient for epitope presentation. Here, we demonstrated the applicability of an FFV infection-based vaccine epitope delivery strategy using Bet-Ova FFV vectors.

To determine whether EL4 and RMA cells are at least partially susceptible to FFV infection, pCF-7 and different pCF-Bet-Ova vector stocks were prepared by transfection of 293T cells. Culture supernatants containing released viral particles were collected 2 d p.t. and were used to infect EL4 or RMA target cells for four days. In parallel, transduced 293T cells were used for co-cultivation with EL4 target cells. Both sets of target cells were analyzed in IFNγ ELISpot assays.

**Fig 8:**
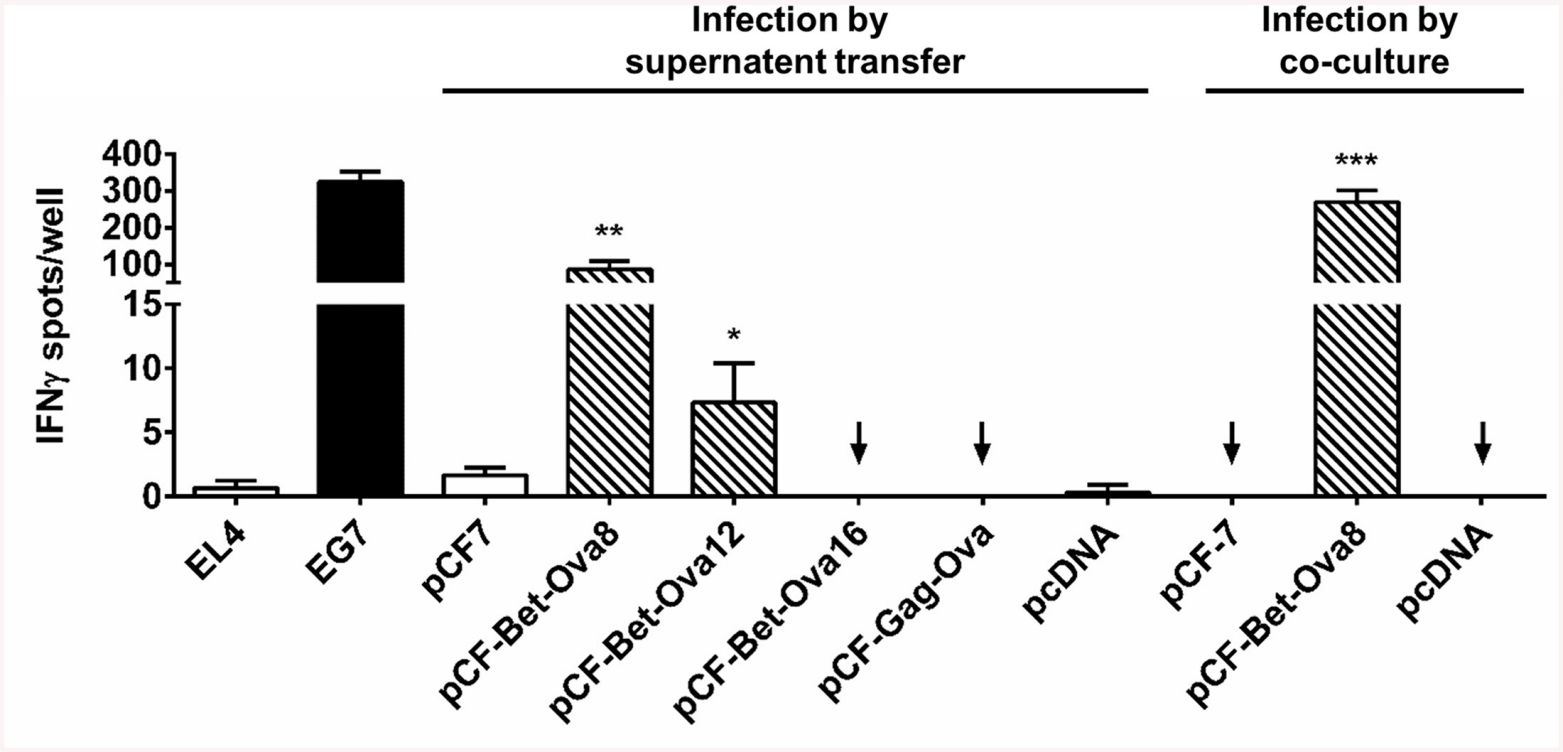
Target cells infected by FFV expressing Bet-SIINFEKL vectors stimulate SIINFEKL-specific CTLs. For infection by supernatant transfer, 293T cells were transfected with pCF-7, pCF-Bet-Ova8, pCF-Bet-Ova12, pCF-Bet-Ova16, pCF-Gag-Ova, or pcDNA. Two d p.t., culture supernatants were cleared and applied onto EL4 cells. For infection by co-culture, 293T cells transfected with pCF-7, pCF-Bet-Ova8, or pcDNA were cultured together with EL4 cells 2 d p.t. Thereafter, cells were harvested and co-cultured with OVA-specific CTLs in ELISpot assays. Plates were probed after 24 h of co-cultivation for the presence of secreted IFNγ. The stable OVA-expressing cell line EG7 served as positive control (black bar). Untransfected EL4, EL4 transfected with pCF7 or with pcDNA were used as negative controls (white bars). Results are shown as number of IFNγ spots per 5000 CTLs/well. Values represent a single experiment performed in triplicate. * represents a p-value of less than 0.05 when compared to pCF-7; **, p-value < 0.01; ***, p-value < 0.001.

EL4 cells cultured together with FFV-Bet-Ova8-containing supernatant strongly induced IFNγ secretion from OVA-specific CTLs. IFNγ production was even stronger when EL4 cells were co-cultured together with FFV-Bet-Ova8-releasing 293T cells (Fig 8). FFV-Bet-Ova12 still induced a statistically significant CTL response in co-cultures, whereas no reactivity was observed with the parental or empty vectors or the Gag-OVA chimera pCF-Gag-Ova. These data show that SIINFEKL is processed from Bet-Ova8 and -12 within infected cells. Insertion of SIINFEKL and its flanking sequences into Bet at a site further upstream of the C-terminus, as with FFV Bet-Ova16, interfered either with EL4 target cell infection or with efficient epitope processing.

**Fig 9:**
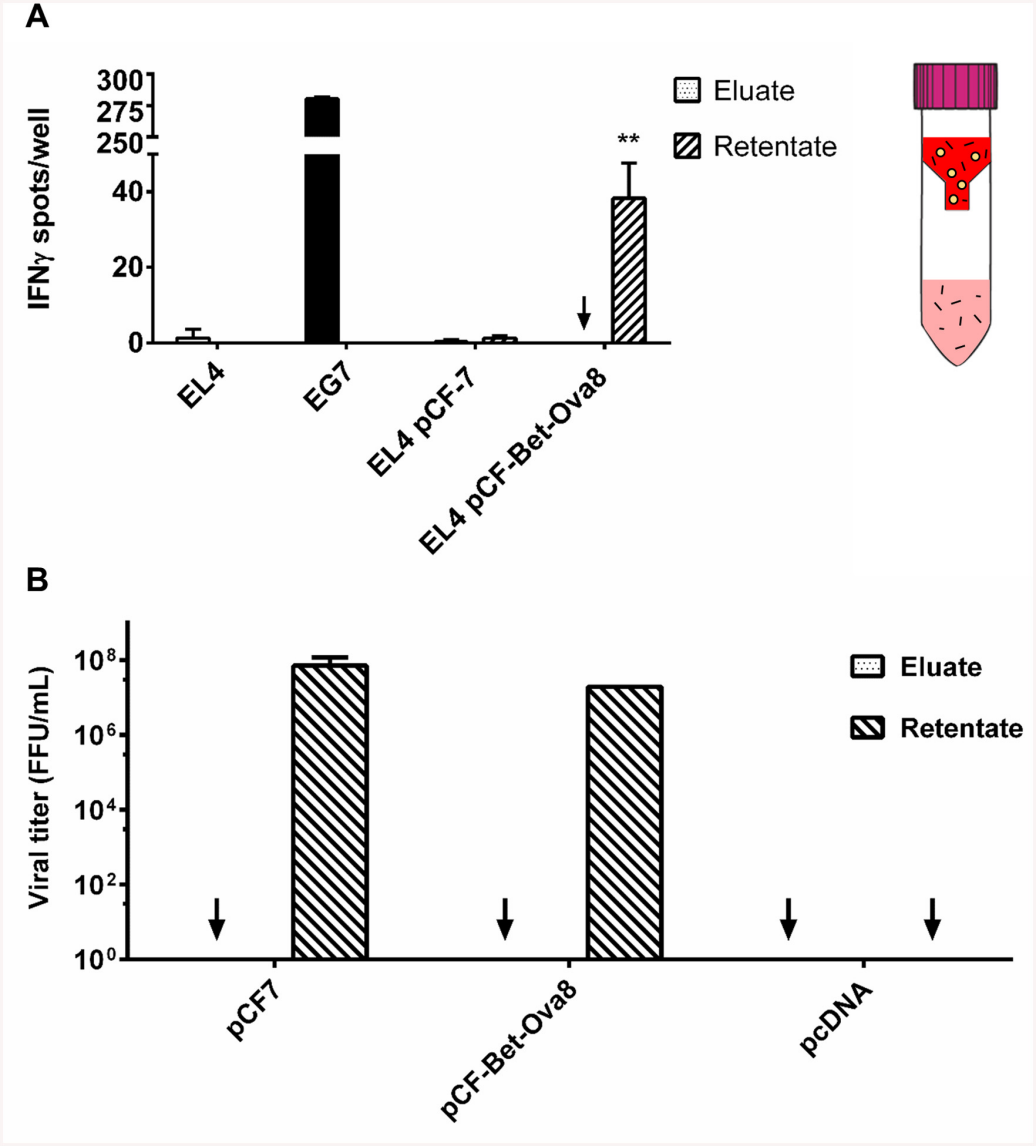
Activation of SIINFEKL-specific CTL by pCF-Bet-Ova8 infected EL4 cells is not due to external peptide loading. HEK293T cells were transiently transfected with pCF-7 or pCF-Bet-Ova8. After 2 d, cell culture supernatants were harvested, cleared, and passed through a 100-kDa centrifugal filter. A) The filtrate (dotted bars) and retentate (striped bars) fractions were applied onto EL4 cells. After 4 d, EL4 cells were harvested and co-cultured with OVA-specific CTLs in an ELISpot assay. Plates were probed after 24 h of co-cultivation for the presence of secreted IFNγ. EG7 cells were used as positive control (black bar). EL4 cells, either untreated or infected with wild-type FFV, and corresponding filtrate fractions were used as negative controls (white bars). Results are shown as number of IFNγ spots per 12500 CTLs/well. ** represents a p-value of less than 0.01 when compared to pCF-7. B) Viral titers were determined for the retentate and filtrate fractions, confirming absence of viral particles in the filtrates.

To exclude that IFNγ secretion from OVA-specific CTLs is the result of external loading with peptides or proteins from culture medium used as inoculum, supernatants from 293T cells producing FFV vectors were fractionated for subsequent individual analyzes. Supernatants from 293T cells transfected with pCF-7 and pCF-Bet-Ova8, which contained released viral particles, were collected 2 d p.t. and passed through a 100-kDa filter to retain intact viral particles. The retentate, containing viral particles, and the filtrate, containing proteins and peptides released from the cells, were used to treat EL4 target cells. EL4 cells infected with the retentate were able to activate CTLs (32–49 IFNγ spots/well), while the same cells incubated with the filtrate showed no ability to activate CTLs (0–3 IFNγ spots/well) (Fig 9A). The absence of viral particles in the filtrate was confirmed by addition of the retentate and the filtrate, respectively, to FeFAB cells (Fig 9B). This result indicates that OVA-specific CTL activation was due to viral entry into target cells, intracellular antigen processing and MHC-I-restricted presentation of the OVA-specific CTL epitope.

## Discussion

Immunotherapy of persistent infections and chronic diseases like HIV/AIDS and cancer represents one of the most promising and rapidly expanding avenues of clinical therapy. For therapeutic vaccination and immunotherapy, unconventional and novel adjuvants and epitope delivery platforms are needed to induce efficient clearance of persistent pathogens or of cancer cells originating from autologous tissue. Here, replicating viruses, which induce strong innate and adaptive immune responses and long-lasting memory, are excellent vehicles for antigen expression and epitope presentation. Retroviruses are especially well-suited for this purpose because they are highly immunogenic and can persist in the host via genome integration, enabling persistent antigen expression and presentation (20). In contrast to other retroviruses, the members of the FV subfamily (*Spumaretrovirinae*) are considered apathogenic and may be safer alternatives compared to lenti- and gammaretrovirus-based vectors [22]. Previous studies using FVs in gene therapy and vaccination approaches have paved the way for further development of FVs as vector platforms [17,72].

This study explores the suitability of replicating FFV vaccine vectors for B and T cell epitope presentation. The epitopes tested in this exploratory study were inserted by replacing viral sequences to minimize disruptions to the vector backbone. Epitopes were selected for their disease relevance, predicted risk of secondary structure disruption, and the availability of biological tools for analysis. One strategy was dependent on the untargeted, unbiased, and bioinformatics-based identification of sequences within the five FFV proteins similar to known B and T cell epitopes and their subsequent incorporation into the identified sites. The second strategy was based on modification of domains previously identified to tolerate changes with no or minor impact on virus replication. The success of these two strategies was evaluated by investigating protein expression, particle release, and the ability to sustain productive infection within two permissive host cell lines. Overall, both methods showed similar success rates.

However, the choice of the viral protein serving as scaffold for the epitope insertion was decisive, as our results clearly show that Bet is a much more flexible scaffold for T cell epitopes than structural proteins such as Gag or Env. Viruses with epitope incorporations into the C-terminus of Bet or based on sequence homology were more likely to remain functional, as Bet is not a structural protein involved in particle release. In addition, Bet is under positive evolution as a viral protein counteracting intrinsic immune attack of the host [73]. Thus, it must rapidly adapt to host counter-defense factors and should be intrinsically flexible. A previous study showed that the C-terminus of Bet is not conserved and can be replaced by related sequences [58].

The initial aim of the study was to explore the function of fully or partially RC FFV-based vectors and their potential use for immunization. The observation that even non-replicating FFV vaccine vectors efficiently stimulated specific CTL responses *in vitro* strongly suggests that strong replication is not a prerequisite for functional CTL recognition. Since FVs are capable of host genome integration and prolonged protein expression, they can constantly provide epitopes for MHC-I-restricted presentation.

As virus-mediated vaccine delivery activates innate immunity, attenuated or fully replicating FV are likely to deliver the adjuvant effects needed to establish protective or even therapeutic immune responses. Though soluble peptides or proteins such as Bet could be capable of inducing CTL responses, the immunological memory established by the adaptive immune system would likely be much weaker. In addition, highly ordered structures, such as viral particles, displaying repetitive B cell epitopes are recognized during immune surveillance more efficiently [2]. A study performed by Mühle et al., for example, employed purified FFV Bet proteins fused to the membrane external proximal region of HIV gp41 [31]. Though HIV-specific antibodies were produced, these antibodies could not protect against HIV-1 infection. However, even if infected cells are cleared upon recognition by specific T cells, RC FV vectors may still be desirable in clinical therapy [74]. They could be used for local vector delivery to target sites poorly accessible by surgical intervention.

The use of live vaccine vectors also has some practical advantages. Small amounts of RC vectors can spread and amplify in the patient, lowering material requirements and production costs. If the RC vector is safe, it can be easily produced by serial passaging in permissive cells, as shown here. Application of RC FV vectors in patients would also reveal novel insights into the immunology of an FV infection. However, RC viral vectors based on pathogenic viruses may revert to the wt; in fact, this is not a concern with FV-based vectors due to their inherent apathogenicity.

T cell epitopes were grafted into Bet and tested for efficient endogenous processing and MHC I-restricted presentation to cognate T cells using epitope-specific CTL lines. Epitope processing and presentation was evaluated independent of the transduction ability of FFV into mouse cells using transfection of sub-genomic Bet expression constructs. The insertion of three CTL epitopes, one derived from a model antigen OVA and two originating from clinically relevant tumor antigens (TRP-2, and HPV16E7) showed efficient processing from the C-terminus of FFV Bet. The results using these three T cell epitopes are the first characterization of T cell epitope generation and presentation using FVs of any species. PFV Bet was also explored as a potential scaffold for epitope incorporation. The HPV16E7 T cell epitope incorporated into the C-terminus of PFV Bet was successfully processed and presented to MHC I-restricted CTLs. Importantly, sensitization of the infected target cells for CTL recognition by external loading of MHC- I molecules with unspecific cleavage products or released peptides could be excluded (Fig 9).

Proteases involved in CTL epitope generation are thought to prefer cleavage behind specific residues [75]. However, the underlying requirements are not comprehensively defined by now, making accurate prediction of proper T cell epitope cleavage from the precursor forms to a certain degree speculative. In this study, the presence/absence or composition of flanking sequences surrounding the H2-K^b^-restricted CTL epitope SIINFEKL did not seem to interfere with epitope processing, at least when the epitope was engineered into the C-terminus of Bet. This also confirms previous studies showing that SIINFEKL itself appears to be protected from proteolytic cleavage [76]. The TRP-2- and HPV16E7-specific CTL epitopes were also efficiently processed from the C-terminus of Bet.

Although there is a growing list of well-defined CTL epitopes that could be useful for immunotherapy, many of them, in particular MHC II-restricted CD4+ T cell epitopes, have a size larger than nine amino acids, thus being probably more disruptive when integrated into viral proteins [77,78]. The epitope sequences themselves can also be optimized by modifying individual residues, particularly at the anchor positions within the epitope. For example, within the MHC I restricted system, variants of the TRP-2-specific CTL epitope containing methionine instead of valine at the N-terminal anchor position were shown to improve stability of the MHC-I-peptide complex, thereby promoting improved immunogenicity [79].

This is the first study demonstrating MHC I-restricted T cell epitope presentation from FV, raising the obvious question whether FVs are suitable delivery vectors for foreign epitopes for T cell stimulation *in vivo*. So far, *in vitro* studies showed up regulation of MHC I surface expression in PFV-infected cells, though this was IFNγ-independent [80]. Moreover, a recent study showed type I IFN secretion upon FV infection *in vitro* and the responsiveness of FV replication towards different IFNs [81]. There is no evidence on the effect of FV infection on type II IFN (IFNγ) expression. It is also currently unknown whether and to which degree FV-specific CTLs could influence virus replication and persistence *in vitro*. The data presented here using the cloned FFV-FUV isolate, which is fully RC in experimentally infected cats [16], indicate that CTL responses are (at least) not fully abrogated by FFV proteins. Apparently, FFV and PFV Bet do not interfere with CTL recognition of the murine thymoma cell line EL-4. It is unclear whether other FV proteins or the recently identified FV-encoded miRNAs [82] have immune-suppressive functions. Such viral immune evasion effectors may have reduced CTL reactivity to FFV-Bet-OVA-8-transduced target cells. However, we believe this was mainly due to limited permissiveness of murine EL-4 cells toward transduction by a feline virus, since FV infection has been described to increase MHC-I expression [80].

Vaccines based on only one antigenic epitope may be rendered non-functional or ineffective when a single residue in the epitope is changed. Viruses generally mutate much faster than the host, a strategy that viruses exploit to evade immune detection [83,84]. This high mutation rate complicates the development of antiviral therapies and vaccines based on single epitopes or target antigen, as exemplified in the case of HIV. The same characteristics are embodied by cancer cells, which have a high mutation rate due to the dysfunction of several pathways including such involved in cell cycle control. The use of multiple epitopes of the same target protein would help to overcome MHC-I polymorphism and prevent immune escape.

Our studies have shown that the C-terminus of Bet is amenable to modification by small peptides, such as MHC-I-restricted CTL epitopes [31,58]. Different epitopes could be inserted into this region. Our studies as well as the in vivo and in vitro data by Schwantes et al. [16,17] indicate that a size of 15 amino acids should allow maintenance of replication as well as stable maintenance and expression of the heterologous sequences. Although not experimentally tested here, molecular characterization of the genetic stability of the replicating vectors is mandatory in future animal experiments. A panel combining multiple T or B cell epitopes, each cloned into a different vector using the same strategy, would bypass the insert size constraints seen in Schwantes *et al*. [16,17]. Such a panel of viruses could be applied as a mixture into the patient. Alternatively, the six epitopes derived from related proteins of the MAGE tumor antigen family [85] and tested individually in Bet (Fig 2) could be also combined in a single vector provided that Bet function is still maintained at a sufficient level. MAGE proteins are involved in cell cycle progression, have been shown to be up-regulated in melanoma, and are currently explored as targets in immunotherapy approaches [65]. Mixtures of several of these T cell epitopes within one therapeutic vaccine should enable destruction of melanoma cells expressing any member of the protein family, thus limiting the development of resistant tumor escape variants, or the need for multiple therapeutic targets. Previous studies [16,17] exploring the use of FFV as a vector for large inserts found that inserts of more than 100 amino acids in the C-terminus of Bet were stable for more than ten weeks. Given the comparatively small size of the inserts used in this study, we do not expect any great mutation pressure on our epitopes. Moreover, FVs are also thought to have a much lower mutation rate than other retroviruses [86]. However, the inevitable occurrence of a random mutation would not render the vector ineffective. Such mutations might even allow the vector to evolve alongside the cancer cell genome as it seeks to evade immune detection by mutating targeted epitopes.

Realistically, the use of integrating RC vectors, even those based on apathogenic viruses such as FVs, would be restricted to the treatment of diseases where the benefit of immunotherapy greatly outweighs the risk of insertional mutagenesis and wt reversion. FVs do not appear to have a propensity for transcriptionally active regions, which is observed with other lentiviruses [19]. As the integration site is still random and cannot be definitively predetermined, there is still a risk of insertional mutagenesis, which must be further characterized. The clinical application of RC FV vectors will require further study of the clinical presentation and the pathways involved in FV infection. A profound understanding of the immune reaction in response to FV infection will be critical in order to define the risks associated with FVs and to determine how RC FV vectors can be used as epitope scaffolds. The lack of documentation of FV-associated disease, despite known infected populations, indicates that FVs are relatively safe vectors. However, a risk-benefit analysis would need to be performed on an individual basis.

FV-based vaccine vectors continue to appear promising as new viral vectors for gene therapy and therapeutic vaccination. The *in vitro* data presented here must be validated by *in vivo* studies. Since PFV appears to infect mouse cells efficiently, the model epitopes used in our study should be transferred into a PFV proviral backbone. To test whether epitope-specific CTLs can be activated and expanded upon exposure to the vector, CTLs should be isolated from immunized mice and tested for specificity using IFNγ ELISpot assays. Cytokine profiling of immune cells from immunized mice would not only confirm specific CTL activation but could also shed light on the innate and adaptive immune responses against the FV vector itself. Tumors expressing the epitope could be transplanted into immunized mice and the extent of tumor growth compared to a non-immunized control group could be monitored, allowing quantitative evaluation to FV-mediated tumor protection. The work presented here opens a new chapter in FV vector development. The studies in FFV need to be translated into the more promiscuous PFV, which is a potential vector in both mice and humans. By exploring T cell epitope presentation from FV proteins, the present study provides a first glimpse into the utility of FVs as a vector for the ultimate goal of human immunotherapy.

## Supporting Information

S1 Fig A) Cell lysates and B) enriched culture supernatants of 293T cells transfected with modified provirus were analyzed by immunoblotting. Cells and supernatants were harvested 2 d p.t. and probed using polyclonal sera against Gag MA and Env TM. Released particles in which Env and Gag are both detectable may be whole virus. Particles containing Env only, as seen in most recombinants, are non-infectious Env SVPs. The Gag precursor (p52), cleaved mature Gag (p48), Env precursor (gp130Env), mature TM (gp48TM) and a cell lysate-associated TM isoform (TM^CL^) are indicated by arrows. Proper protein loading of cell lysates was determined by probing for β-actin. C) Syncytia formation in 293T cells transfected with recombinant proviruses. Red frames indicate attenuated or defective syncytia formation.
(TIF)

S2 Fig A) Cell lysates and B) enriched culture supernatants of 293T cells transfected with modified provirus were analyzed by immunoblotting. Cells and supernatants were harvested 2 d p.t. and probed using polyclonal sera against Gag MA and Env TM. Released particles in which Env and Gag are both detectable may be whole virus. Particles containing Env only, as seen in most recombinants, are non-infectious Env SVPs. The Gag precursor (p52), cleaved mature Gag (p48), Env precursor (gp130Env), mature TM (gp48TM) and a cell lysate-associated TM isoform (TMCL) are indicated by arrows. Proper protein loading of cell lysates was determined by probing for β-actin.
(TIF)

S1 Table Primers used in this study. (DOCX)

S2 Table Plasmids constructed in this study. (DOCX)
