# Supplementary figures and images for: Replication-Competent Foamy Virus Vaccine Vectors as Novel Epitope Scaffolds for Immunotherapy
Source: PLoS One. 2015 Sep 23;10(9):e0138458. doi: 10.1371/journal.pone.0138458 (PMC4580568; doi:10.1371/journal.pone.0138458)

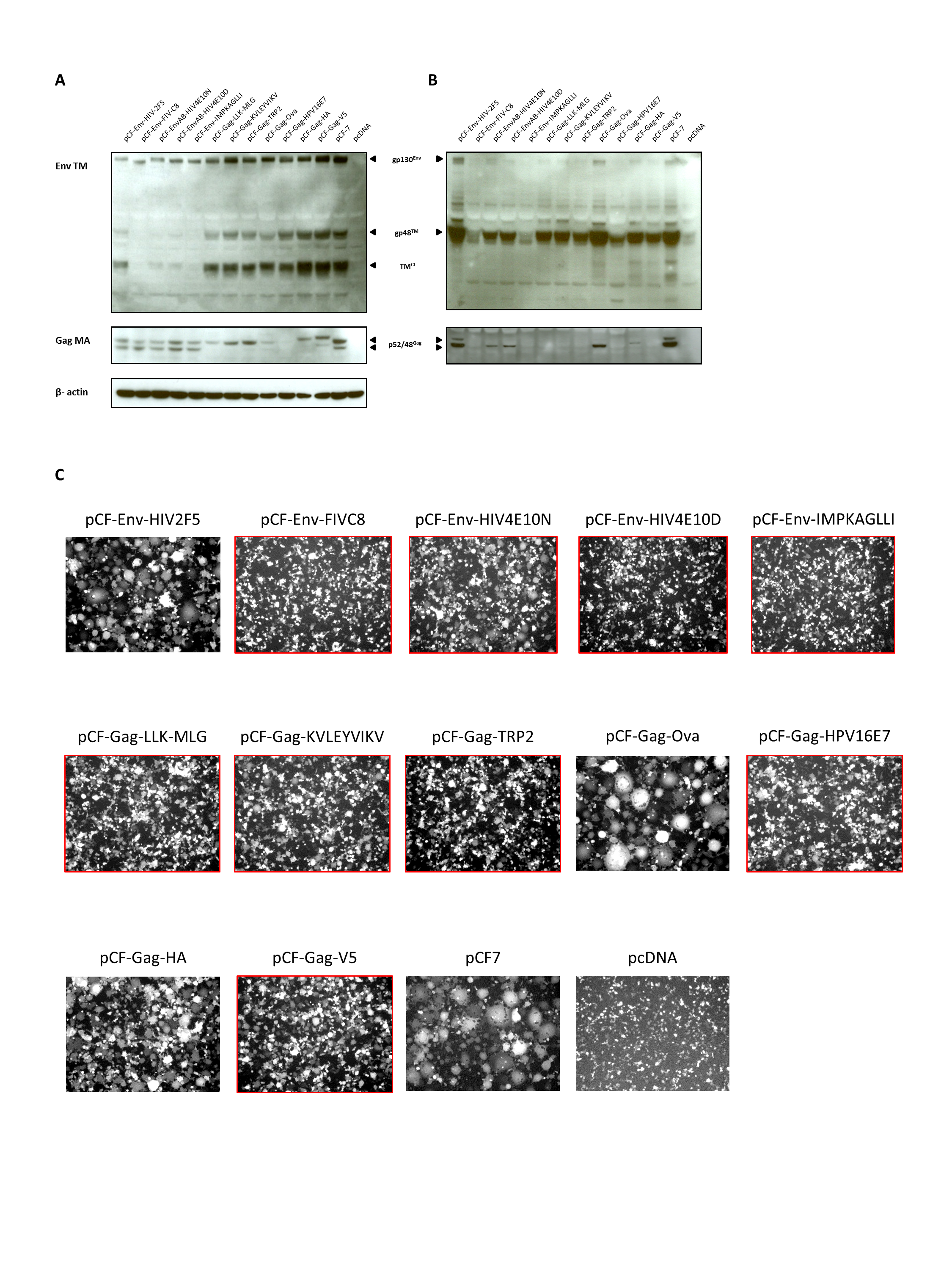

Supplement: S1 Fig — A) Cell lysates and B) enriched culture supernatants of 293T cells transfected with modified provirus were analyzed by immunoblotting. Cells and supernatants were harvested 2 d p.t. and probed using polyclonal sera against Gag MA and Env TM. Released particles in which Env and Gag are both detectable may be whole virus. Particles containing Env only, as seen in most recombinants, are non-infectious Env SVPs. The Gag precursor (p52), cleaved mature Gag (p48), Env precursor (gp130Env), mature TM (gp48TM) and a cell lysate-associated TM isoform (TMCL) are indicated by arrows. Proper protein loading of cell lysates was determined by probing for β-actin. C) Syncytia formation in 293T cells transfected with recombinant proviruses. Red frames indicate attenuated or defective syncytia formation. (TIF) [file pone.0138458.s001.tif]

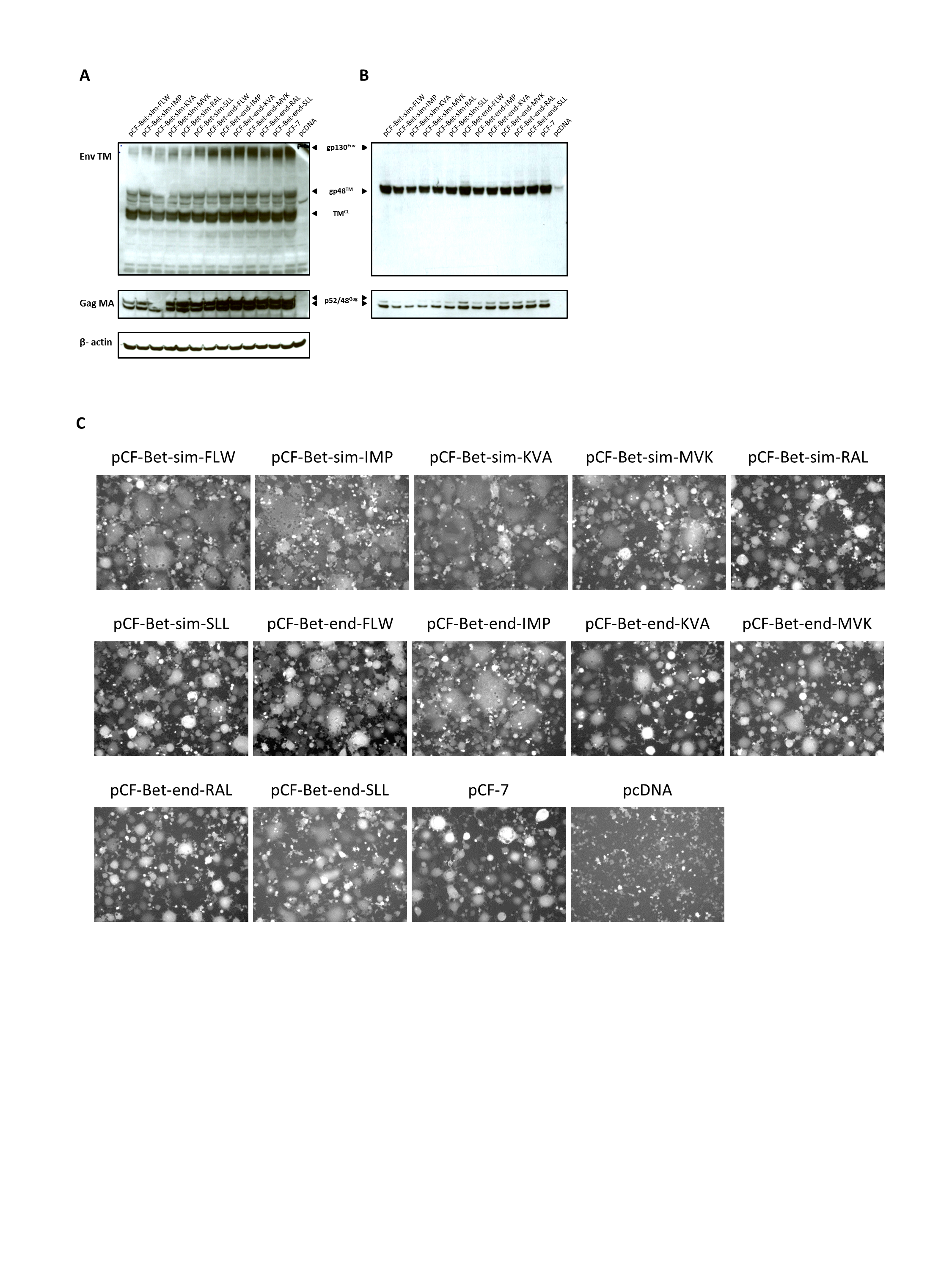

Supplement: S2 Fig — Cells and supernatants were harvested 2 d p.t. and probed using polyclonal sera against Gag MA and Env TM. Released particles in which Env and Gag are both detectable may be whole virus. Particles containing Env only, as seen in most recombinants, are non-infectious Env SVPs. The Gag precursor (p52), cleaved mature Gag (p48), Env precursor (gp130Env), mature TM (gp48TM) and a cell lysate-associated TM isoform (TMCL) are indicated by arrows. Proper protein loading of cell lysates was determined by probing for β-actin. (TIF) [file pone.0138458.s002.tif]
