# Supplementary material for: Replication-Competent Foamy Virus Vaccine Vectors as Novel Epitope Scaffolds for Immunotherapy
Source: PLoS One. 2015 Sep 23;10(9):e0138458. doi: 10.1371/journal.pone.0138458 (PMC4580568; doi:10.1371/journal.pone.0138458)
Supplement: S1 Table — (DOCX) [file pone.0138458.s003.docx]

| Primer name | Sequence (5'-3') |
| --- | --- |
| B- and T-cell epitopes in pCF-7 Gag and Env | |
| FFV Gag F | GAGGAAGGATGGCTCGAGA |
| FFV Gag R | GGTCTCGGCTGATTTAGTTG |
| FFV Env A F | GGATGGAATGGAATGCTCAC |
| FFV Env A R | GGTTGTTGAATCCACACCTTA |
| FFV Env B F | GTAATTGGTATCCACGTGATCT |
| FFV Env B R | TGCAGGACGAGTAGGATCC |
| FFV Env B HIV 2F5 F | AGAGCTGGATAAGTGGGCCCAGCTCCTTGGAGAAGCAA |
| FFV Env B HIV 2F5 R | GGGCCCACTTATCCAGCTCTCCTTCGTGAAGGTCCAAG |
| FFV Env B HIV C8 F | TGGGAGGATTGGGTCGGATGGATCGCATATACTGACTCTTTTCCAT |
| FFV Env B HIV C8 R | GATCCATCCGACCCAATCCTCCCAATTCTCAGTTCCATCATACAAA |
| FFV Env A HIV 4E10 N F | TTGGTTCAACATCACCCTACCTTCTTCGGTACAACAA |
| FFV Env A HIV 4E10 N R | GGTAGGGTGATGTTGAACCAATTACTATATAGTGCATCA |
| FFV Env A HIV 4E10 D F | TTGGTTCGACATCACCCTACCTTCTTCGGTACAACAA |
| FFV Env A HIV 4E10 D R | GGTAGGGTGATGTCGAACCAATTACTATATAGTGCATCA |
| FFV Env B IMPKAGLLI F | TTATGCCAAAGGCAGGATTATTAATAAATAAAACATATTATACTTTCTC |
| FFV Env B IMPKAGLLI R | TTAATAATCCTGCCTTTGGCATAATACAGATATCATATGGGTTAATA |
| FFV Gag LLQ_MLG F | AAGAGAGCCGGTAACAAAGGCTGAGATGCTTGGCTCGGTTATTGGCAATACTCCA |
| FFV Gag LLQ_MLG R | CCTTTGTTACCGGCTCTCTTGCCCTGTATTTTAGTAAAACTGCTCCTGGTATAG |
| FFV Gag KVLEYVIKV F | GTTCTAGAATACGTTATTAAGGTAAGGGTAGTTAATGCCTTAGTA |
| FFV Gag KVLEYVIKV R | TTAATAACGTATTCTAGAACCTTAAATACTCCTTCAATAGCGGC |
| FFV Gag Trp2 F | TCGGTCTACGATTTCTTCGTGTGGCTTGGAAGATCTACAGC |
| FFV Gag Trp2 R | CACGAAGAAATCGTAGACCGATGGTGGAGTATTGCCAATAAC |
| FFV Gag Ova F | AGCATAATAAATTTCGAGAAGCTGATCTTGATAGCAGGGCCATAT |
| FFV Gag Ova R | CAGCTTCTCGAAATTTATTATGCTAGGTTCCAAATCATATCCAGG |
| FFV Gag HPV16 F | GCCCACTATAACATCGTGACCTTCGCCTTTTTGGACTTAGAGCC |
| FFV Gag HPV16 R | GTCACGATGTTATAGTGGGCCCGTATCAAGATATCCTCTCTCAAAT |
| FFV Gag HA F | CCCTTACGACGTGCCTGACTACGCTACGCGTAGACCTATACCAGGAGCAGT |
| FFV Gag HA R | AGTCAGGCACGTCGTAAGGGTAGCGGCCGCCACCCACACCTCCAGCTTGT |
| FFV Gag V5 F | TCCCTAACCCTCTGCTGGGCCTGGACAGCACCACGCGTAGACCTATACCAGGAGCAGT |
| FFV Gag V5 R | GCCCAGCAGAGGGTTAGGGATAGGCTTGCCGCGGCCGCCACCCACACCTCCAGCTTGT |
|  |  |
| T-cell epitopes in pCF-7 Bet (similarity vs. position) | |
| FFV Bet F | CTTATGAGAAGGCATGTGCAA |
| FFV Bet R | GGCTCTAGATGTAAGACGGT |
| R2 Bet sim IMP F | ATGCCTAAGGCCGGCCTGCTGATCATCGCCGCTACACTTACTAAA |
| R2 Bet sim IMP R | CAGCAGGCCGGCCTTAGGCATGATCAATTCTCTAGTTAGCATAGTC |
| R2 Bet end IMP s | CCGGTGATCATGCCTAAGGCCGGCCTGCTGATCA |
| R2 Bet end IMP as | CGCGTGATCAGCAGGCCGGCCTTAGGCATGATCA |
| R2 Bet sim FLW F | CTGTGGGGCCCTCGGGCCCTGGTGCGTAGACATAGGAATCTT |
| R2 Bet sim FLW R | CAGGGCCCGAGGGCCCCACAGGAAATCATGTTCAGGATCAAGACT |
| R2 Bet end FLW F | TGTGGGGCCCTCGGGCCCTGGTGACGCGTGCAACATCTGAGTCATCTGA |
| R2 Bet end FLW R | CCAGGGCCCGAGGGCCCCACAGGAACACCGGTTTTAGCTCAGGATCACA |
| R2 Bet sim MVK F | GTGAAGATCTCCGGCGGCCCTAGAACACAGCAATGTATTATGATGAA |
| R2 Bet sim MVK R | AGGGCCGCCGGAGATCTTCACCATCCAAATGAGAAAGGGTCTCAT |
| R2 Bet end MVK s | CCGGTGATGGTGAAGATCTCCGGCGGCCCTAGAA |
| R2 Bet end MVK as | CGCGTTCTAGGGCCGCCGGAGATCTTCACCATCA |
| R2 Bet sim KVA F | GTGGCCGAGCTGGTGCACTTCCTGATATTTGGAAGTGCCTCTGG |
| R2 Bet sim KVA R | GAAGTGCACCAGCTCGGCCACCTTGACTCTGGGATACAGGGAA |
| R2 Bet end KVA s | CCGGTGAAGGTGGCCGAGCTGGTGCACTTCCTGA |
| R2 Bet end KVA as | CGCGTCAGGAAGTGCACCAGCTCGGCCACCTTCA |
| R2 Bet sim SLL F | AGCCTGCTGATGTGGATCACCCAGTGCCAACAAAAGCTCTTT |
| R2 Bet sim SLL R | GGTGATCCACATCAGCAGGCTCTCTCTTTCTGACATTTCTTCT |
| R2 Bet end SLL s | CCGGTGAGCCTGCTGATGTGGATCACCCAGTGCA |
| R2 Bet end SLL as | CGCGTGCACTGGGTGATCCACATCAGCAGGCTCA |
| R2 Bet sim RAL F | GCCCTGGCCGAGACCAGCTACGTGGAATAGAAAGCCTGAATTTACC |
| R2 Bet sim RAL R | GTAGCTGGTCTCGGCCAGGGCTCTATCCAAGAATTTCAATTCCTGC |
| R2 Bet end RAL s | CCGGTGAGAGCCCTGGCCGAGACCAGCTACGTGA |
| R2 Bet end RAL as | CGCGTCACGTAGCTGGTCTCGGCCAGGGCTCTCA |
|  |  |
| T-cell epitopes in pCF-7 Bet (OVA) | |
| Ova8 F | TAATCAACTTTGAGAAACTGTAGAAAGCCTGAATTTACCT |
| Ova8 R | TTTCTCAAAGTTGATTATACTTGCCCTATCCAAGAATTTC |
| Ova12 F | AGTATAATCAACTTTGAGAAACTGACTGAATAGAAAGCCTGAATT |
| Ova12 R | TCTCAAAGTTGATTATACTCTCAAGGAATTTCAATTCCTGCAATA |
| Ova16 F | ATAATCAACTTTGAGAAACTGACTGAATGGACCTAGAAAGCCTGAATTTACCT |
| Ova16 R | AGTTTCTCAAAGTTGATTATACTCTCAAGCTGCTCCTGCAATAATGGTTTTAG |
| Ova20 F | TATAATCAACTTTGAGAAACTGACTGAATGGACCAGTTCTTAGAAAGCCTGAATTTACCT |
| Ova20 R | GTTTCTCAAAGTTGATTATACTCTCAAGCTGCTCAAGGCCTTTTAGCTCAGGATCACAGG |
| Ova20C5 F | ATAATCAACTTTGAGAAACTGACTGAATGGACCAGTTCTTCATCTGACTCTGAATAGAA |
| Ova20C5 R | GTTTCTCAAAGTTGATTATACTCTCAAGCTGCTCAAGGCCACAGGCTATGAATTTTCTT |
| Ova20C10 F | TATAATCAACTTTGAGAAACTGACTGAATGGACCAGTTCTAGGGCAACATCTGAGTCAT |
| Ova20C10 R | TTTCTCAAAGTTGATTATACTCTCAAGCTGCTCAAGGCCTCTTCCATCAGGAAGTATCA |
| Ova20C15 F | TATAATCAACTTTGAGAAACTGACTGAATGGACCAGTTCTTTGAAATTCTTGGATAGGGC |
| Ova20C15 R | GTTTCTCAAAGTTGATTATACTCTCAAGCTGCTCAAGGCCTATCACTTTTGTTCCTTTAG |
| Ova20C20 F | ATAATCAACTTTGAGAAACTGACTGAATGGACCAGTTCTCCATTATTGCAGGAATTGAA |
| Ova20C20 R | AGTTTCTCAAAGTTGATTATACTCTCAAGCTGCTCAAGGCCTTTAGGAAATTTAATATT |
|  |  |
| T-cell epitopes in FFV Bet expression vector | |
| Betmax-NheI F | GCGCTAGCATGGCTTCAAAATACCCGGA |
| Ova8-XhoI R | CGCGCTCGAGCTACAGTTTTTCAAAGTTGATT |
| Ova12-XhoI R | CGCGCTCGAGCTATTCAGTCAGTTTTTCAAAG |
| Ova16-XhoI R | CGCGCTCGAGCTAGGTCCATTCAGTCAGTTTTTCAAAGTTGATTATACTCTCAAGCTGCTCCTGCAATAATGGTTTTAG |
| Ova20-XhoI R | CGCGCTCGAGCTAAGAACTGGTCCATTCAG |
| Betmax XhoI R | CCGCTCGAGCTATTCAGAGTCAGATGACTCAG |
| pmaxBet internal F | GGAGAGCACAAGCTGATGAT |
| Bet Trp2 R | CGAGCTCGAGCTACAGCCACACGAAGAAATCGTACACGCTCCTATCCAAGAATTTCAATTCC |
| Bet HPV16 E7 R | CGAGCTCGAGCTAGAAGGTCACGATGTTGTAATGCGCACGCCTATCCAAGAATTTCAATTCC |
|  |  |
| T-cell epitopes in pCF-7 Bet (TRP2 and HPV16E7) | |
| pCF7 Bet-Trp2 F | TGTACGATTTCTTCGTGTGGCTGTAGAAAGCCTGAATTTACCTG |
| pCF7 Bet-Trp2 R | CACACGAAGAAATCGTACACGCTCCTATCCAAGAATTTCAATTCC |
| pCF7 Bet-HPV16 F | CGCATTACAACATCGTGACCTTCTAGAAAGCCTGAATTTACCTG |
| pCF7 Bet-HPV16 R | GTCACGATGTTGTAATGCGCACGCCTATCCAAGAATTTCAATTCC |
|  |  |
| T-cell epitope in PFV Bet expression vector (HPV16E7) | |
| PFV Bet NheI F | GCGGCTAGCATGGATTCCTACGAAAAAGAAG |
| PFV Bet XhoI R | CCGCTCGAGTTAGAAGGGTCCATCTGAGT |
| PFV Bet add XhoI HPV16E7 R | CCGCTCGAGTTAGAAGGTCACGATGTTGTAATGCGCACGGAAGGGTCCATCTGAGTCA |
| PFV Bet XhoI HPV16 R | CCGCTCGAGTTAGAAGGTCACGATGTTGTAATGCGCACGGTCAATGAAGGGGTCACAAT |
