# Supplementary material for: Replication-Competent Foamy Virus Vaccine Vectors as Novel Epitope Scaffolds for Immunotherapy
Source: PLoS One. 2015 Sep 23;10(9):e0138458. doi: 10.1371/journal.pone.0138458 (PMC4580568; doi:10.1371/journal.pone.0138458)
Supplement: S2 Table — (DOCX) [file pone.0138458.s004.docx]

| Plasmid Name | Description and Cloning Strategy | Source |
| --- | --- | --- |
| pCF-7 | FFV-FUV provirus | [104] |
| pcDNA3.1 | Empty vector | Invitrogen |
| pBC-Env | FFV env expressed from pBC12 with CMV-IE promoter | [124] |
| pBC-FFVBet | FFV Bet expressed from pBC12 with CMV-IE promoter | [50] |
| pCF-BBtr | FFV-FUV provirus with truncated bet | [50] |
| pBC-PFV-Bet | PFV Bet expressed from pBC12 with CMV-IE promoter | M. Löchelt |
| pmaxGFP | Pontellina plumata GFP (CopGFP) expressed from CMV-IE promoter | Lonza |
| pCF-7 EnvB HIV 2F5 | pCF-7 with HIV 2F5 epitope inserted into Env, constructed by overlap PCR using AcvI/BspEI and primers EnvB F, EnvB R, EnvB 2F5 F, EnvB 2F5 R | This study |
| pCF-7 EnvB FIV C8 | pCF-7 with FIV C8 epitope inserted into Env, constructed by overlap PCR using AcvI/BspEI and primers EnvB F, EnvB R, EnvB C8 F, EnvB C8 R | This study |
| pCF-7 EnvAB HIV 4E10N | pCF-7 with HIV 4E10 epitope (N-variant, NWFNIT) inserted into Env, constructed by overlap PCR using SalI/BspEI and primers EnvA F, EnvB R, EnvAB 4E10N F, EnvAB 4E10N R | This study |
| pCF-7 EnvAB HIV 4E10D | pCF-7 with HIV 4E10 epitope (D-variant, NWFDIT) inserted into Env, constructed by overlap PCR using SalI/BspEI and primers EnvA F, EnvB R, EnvAB 4E10D F, EnvAB 4E10D R | This study |
| pCF-7 EnvB IMPKAGLLI | EnvB F, EnvB R, EnvB IMP F, EnvB IMP RpCF-7 with MAGE epitope inserted into Env, constructed by overlap PCR using AcvI/BspEI and primers | This study |
| pCF-7 Gag LLK_MLG | pCF-7 with MAGE epitope inserted into Gag, constructed by overlap PCR using XhoI/XmaI and primers Gag F, Gag R, Gag LLK F, Gag LLK R | This study |
| pCF-7 Gag KVLEYVIKV | pCF-7 with MAGE epitope inserted into Gag, constructed by overlap PCR using XhoI/XmaI and primers Gag F, Gag R, Gag KVL F, Gag KVL R | This study |
| pCF-7 Gag Trp2 | pCF-7 with Trp2 epitope inserted into Gag, constructed by overlap PCR using XhoI/XmaI and primers Gag F, Gag R, Gag Trp2 F, Gag Trp2 R | This study |
| pCF-7 Gag Ova | pCF-7 with Ova epitope inserted into Gag, constructed by overlap PCR using XhoI/XmaI and primers Gag F, Gag R, Gag Ova F, Gag Ova R | This study |
| pCF-7 Gag HPV16 | pCF-7 with HPV16 epitope inserted into Gag, constructed by overlap PCR using XhoI/XmaI and primers Gag F, Gag R, Gag HPV16 F, Gag HPV16 R | This study |
| pCF-7 Gag HA | pCF-7 with HA epitope inserted into Gag, constructed by overlap PCR using XhoI/XmaI and primers Gag F, Gag R, Gag HA F, Gag HA R | This study |
| pCF-7 Gag V5 | pCF-7 with V5 epitope inserted into Gag, constructed by overlap PCR using XhoI/XmaI and primers Gag F, Gag R, Gag V5 F, Gag V5 R | This study |
| pCF-7 FLW sim | pCF-7 with MAGE epitope inserted into Bet, constructed by overlap PCR using BspEI/SphI and primers Bet F, Bet R, FLWsim F, FLWsim R | This study |
| pCF-7 IMP sim | pCF-7 with MAGE epitope inserted into Bet, constructed by overlap PCR using BspEI/SphI and primers Bet F, Bet R, IMPsim F, IMPsim R | This study |
| pCF-7 KVA sim | pCF-7 with MAGE epitope inserted into Bet, constructed by overlap PCR using BspEI/SphI and primers Bet F, Bet R, KVAsim F, KVAsim R | This study |
| pCF-7 MVK sim | pCF-7 with MAGE epitope inserted into Bet, constructed by overlap PCR using BspEI/SphI and primers Bet F, Bet R, MVKsim F, MVKsim R | This study |
| pCF-7 RAL sim | pCF-7 with MAGE epitope inserted into Bet, constructed by overlap PCR using BspEI/SphI and primers Bet F, Bet R, RALsim F, RALsim R | This study |
| pCF-7 SLL sim | pCF-7 with MAGE epitope inserted into Bet, constructed by overlap PCR using BspEI/SphI and primers Bet F, Bet R, SLLsim F, SLLsim R | This study |
| pCF-7 FLW end | pCF-7 with MAGE epitope inserted into C-terminus of Bet, constructed by overlap PCR using BspEI/SphI and primers Bet F, Bet R, FLWend F, FLWend R | This study |
| pCF-7 IMP end | pCF-7 with MAGE epitope inserted into C-terminus of Bet, constructed by overlap PCR using BspEI/SphI and primers Bet F, Bet R, IMPend F, IMPend R | This study |
| pCF-7 KVA end | pCF-7 with MAGE epitope inserted into C-terminus of Bet, constructed by overlap PCR using BspEI/SphI and primers Bet F, Bet R, KVAend F, KVAend R | This study |
| pCF-7 MVK end | pCF-7 with MAGE epitope inserted into C-terminus of Bet, constructed by overlap PCR using BspEI/SphI and primers Bet F, Bet R, MVKend F, MVKend R | This study |
| pCF-7 RAL end | pCF-7 with MAGE epitope inserted into C-terminus of Bet, constructed by overlap PCR using BspEI/SphI and primers Bet F, Bet R, RALend F, RALend R | This study |
| pCF-7 SLL end | pCF-7 with MAGE epitope inserted into C-terminus of Bet, constructed by overlap PCR using BspEI/SphI and primers Bet F, Bet R, SLLend F, SLLend R | This study |
| pCF-Bet-Ova8 | pCF-7 with 8 aa Ova epitope in C-terminus of Bet, constructed by overlap PCF using BspEI/SphI and primers Bet F, Bet R, Ova8 F, Ova8 R | This study |
| pCF-Bet-Ova12 | pCF-7 with 12 aa Ova epitope in C-terminus, constructed by overlap PCF using BspEI/SphI and primers Bet F, Bet R, Ova12 F, Ova12 R | This study |
| pCF-Bet-Ova16 | pCF-7 with 16 aa Ova epitope in C-terminus, constructed by overlap PCF using BspEI/SphI and primers Bet F, Bet R, Ova16 F, Ova16 R | This study |
| pCF-Bet-Ova20 | pCF-7 with 20 aa Ova epitope in C-terminus, constructed by overlap PCF using BspEI/SphI and primers Bet F, Bet R, Ova20 F, Ova20 R | This study |
| pCF-Bet-Ova20C5 | pCF-7 with 20 aa Ova epitope 5 aa from C-terminus, constructed by overlap PCF using BspEI/SphI and primers Bet F, Bet R, Ova20C5 F, Ova20C5 R | This study |
| pCF-Bet-Ova20C10 | pCF-7 with 20 aa Ova epitope 10 aa from C-terminus, constructed by overlap PCF using BspEI/SphI and primers Bet F, Bet R, Ova20C10 F, Ova20C10 R | This study |
| pCF-Bet-Ova20C15 | pCF-7 with 20 aa Ova epitope 15 aa from C-terminus, constructed by overlap PCF using BspEI/SphI and primers Bet F, Bet R, Ova20C15 F, Ova20C15 R | This study |
| pCF-Bet-Ova20C20 | pCF-7 with 20 aa Ova epitope 20 aa from C-terminus, constructed by overlap PCF using BspEI/SphI and primers Bet F, Bet R, Ova20C20 F, Ova20C20 R | This study |
| pmaxBet | pmaxGFP with Pontellina plumata GFP (CopGFP) replaced by Bet cDNA, constructed by PCR using NheI/XhoI and primers Betmax NheI F, Betmax R | This study |
| pmaxBet-GFP | pmaxGFP with Bet cDNA C-terminally tagged with CopGFP, constructed by PCR using NheI/XhoI and primers | This study |
| pmaxBet-Ova8 | pmaxBet with 8 aa OVA epitope in C-terminus of Bet, constructed by PCR using NheI/XhoI and primers Betmax NheI F, Ova8-XhoI R | This study |
| pmaxBet-Ova12 | pmaxBet with 12 aa OVA epitope in C-terminus of Bet, constructed by PCR using NheI/XhoI and primers Betmax NheI F, Ova12-XhoI R | This study |
| pmaxBet-Ova16 | pmaxBet with 16 aa OVA epitope in C-terminus of Bet, constructed by PCR using NheI/XhoI and primers Betmax NheI F, Ova16-XhoI R | This study |
| pmaxBet-Ova20 | pmaxBet with 20 aa OVA epitope in C-terminus of Bet, constructed by PCR using NheI/XhoI and primers Betmax NheI F, Ova20-XhoI R | This study |
| pmaxBet-TRP2 | pmaxBet with 9 aa TRP2 epitope in C-terminus of Bet, constructed by PCR using NheI/XhoI and primers Betmax NheI F, TRP2-XhoI R | This study |
| pmaxBet-HPV16E7 | pmaxBet with 9 aa HPV16E7 epitope in C-terminus of Bet, constructed by PCR using NheI/XhoI and primers Betmax NheI F, HPV16E7-XhoI R | This study |
| pCF-Bet-TRP2 | FFV Bet F, FFV Bet R, pCF7 Bet-Trp2 F, pCF7 Bet-Trp2 RpCF-7 with 9 aa TRP2 epitope in C-terminus of Bet, constructed by overlap PCF using BspEI/SphI and primers | This study |
| pCF-Bet-HPV16E7 | pCF-7 with 9 aa HPV16E7 epitope in C-terminus of Bet, constructed by overlap PCF using BspEI/SphI and primers FFV Bet F, FFV Bet R, pCF7 Bet-HPV16 F, pCF7 Bet-HPV16 R | This study |
| pmaxPFVBet | pmaxGFP with CopGFP replaced with PFVBet, constructed by PCR using NheI/XhoI and primers PFV Bet NheI F, PFV Bet XhoI R | This study |
| pmaxPFVBet-HPV16E7 | pmaxGFP with CopGFP replaced with PFVBet containing HPV16E7 epitope replacing 9 aa at C-terminus, constructed by PCR using NheI/XhoI and primers PFV Bet NheI F, PFV Bet XhoI HPV16 R | This study |
| pmaxPFVBet-HPV16E7-add | pmaxGFP with CopGFP replaced with PFVBet containing HPV16E7 epitope added onto C-terminus, constructed by PCR using NheI/XhoI and primers PFV Bet NheI F, PFV Bet add XhoI HPV16E7 R | This study |
